# Anthozoan Chemical Defenses: Integrating Compounds, Enzymatic Activities, and Omics-Based Discoveries

**DOI:** 10.3390/ijms26136109

**Published:** 2025-06-25

**Authors:** Muhammad Zakariya, Oliver J. Lincoln, Isabella D’Ambra, Chiara Lauritano

**Affiliations:** 1 Ecosustainable Marine Biotechnology Department, Stazione Zoologica Anton Dohrn, Via Acton n. 55, 80133 Naples, Italy; zakariyakhan291@gmail.com; 2 School of Biological Sciences, Queen’s University Belfast, 19 Chlorine Gardens, Co. Antrim, Belfast BT9 5DL, UK; olincoln01@qub.ac.uk; 3 Queen’s University Belfast Marine Laboratory, 12-13 The Strand, Co. Down, Portaferry BT22 1PF, UK; 4 Integrative Marine Ecology Department, Stazione Zoologica Anton Dohrn, Villa Comunale, 80121 Naples, Italy; isabella.dambra@szn.it

**Keywords:** bioactive compounds, toxins, omics, enzymes, stress responses

## Abstract

Anthozoa is a species-rich class with an innate immune system that acts as a defensive tool and shares many of its cellular pathways with mammalian immune responses. In addition to immune-related strategies (e.g., allorecognition and xenorecognition), anthozoans have evolved to use compounds or toxins for chemical communication, defense, or predation, which may exhibit biological activities useful for human health, mainly antiviral, antibacterial, anti-inflammatory, anticancer, and antitumor properties of pharmaceutical interest. These compounds/toxins can be alkaloids, amino acids, proteins, ceramides, diterpenes, and sesquiterpenes and are mainly distributed into Hexacorallia and Octocorallia. Anthozoans are enriched in defensive enzymes, which can either be found in anthozoan species or their symbionts and help them survive in hostile conditions. Studies related to genomics and transcriptomics using advanced sequencing efforts revealed the presence of genetic elements in anthozoans that help them survive against abiotic and biotic stressors in the marine environment. This review presents developments and highlights the current state of knowledge about anthozoans’ chemical weaponry that can drive further bioprospection of anthozoan species producing compounds and toxins which may be useful in biotechnological applications. Omics research in Anthozoa is still nascent, and more efforts are required to fully understand the chemical ecology, diversity, and possible biotechnological applications of cnidarian genes and their products.

## 1. Introduction

Cnidarians constitute one of the primitive diverging phyla in the animal kingdom, and knowing about their origin and diversification is crucial to understanding metazoan evolution. Phylum Cnidaria demonstrated divergence between two large clades, namely Anthozoa (corals and sea anemones) and Medusozoa, containing scyphozoans (true jellyfish), cubozoans (box jellies), hydrozoans (hydroids, hydra, and hydromedusae), and staurozoans (stalked medusae) [1,2]. These soft-bodied, mostly sessile organisms have witnessed the emergence (and disappearance) of a large number of life forms since their origin, at least 700 Ma during the Precambrian era [2,3]. Cnidarians have emerged as successful in thriving in all aquatic environments, protecting themselves from predators, remaining efficient in catching prey, and performing other biological roles necessary for survival. These coelenterates (cnidarians) have developed the ability to produce numerous toxins and bioactive molecules as defensive tools and for prey capture [4]. The members of phylum Cnidaria, including jellyfish, sea anemones, and hydrozoans, are all equipped with cnidocytes (nematocytes), which are stinging cells used as chemical arsenals for avoiding predators and capturing their prey [5]. These cells contain an organelle called cnida or cnidocyst, a product of extensive Golgi secretions, which is possibly the most sophisticated organelle in nature, and their explosive secretion is one of the fastest biomechanical reactions documented in the animal kingdom [6,7]. Cnidarians (e.g., hydra and sea anemones) are mainly dependent on allomones which are distributed through the whole organism, both in body tissues and in specialized stinging cells (nematocytes) [8]. Thus, the evolutionary success of cnidarians can be associated with their well-developed defensive and predatory behaviors, particularly their specialized cells and toxins, which have helped them survive and diversify across multiple aquatic environments for millions of years.

Phylogenetic analysis shows that Anthozoa have appeared earlier in evolutionary history than other classes of Metazoa as they possess circular DNA compared to Cubozoa, Scyphozoa, and Hydrozoa, which carry linear DNA [9], a key position in evolution, which highlights their significant ecological role in marine ecosystems and in the food web [10]. Key evolutionary events that have led to the ecological success of anthozoans across the Phanerozoic include modular and colonial form, the ability to deposit a skeleton of crystalline aragonite or calcite, and the well-developed symbiosis with photosynthetic dinoflagellates. These traits support anthozoans in generating biogenic structures, which maintain the whole reef ecosystems in both shallow and deep waters [11].

Anthozoa is considered the most species-rich class with about 12,505 species in the phylum Cnidaria, which are mainly contained in two subclasses: Hexacorallia (8692 species) and Octocorallia (3671 species), as reported by World Register of Marine Species (WoRMS) (www.marinespecies.org, Accessed 8 April 2025). Hexacorallia (also known as Zoantharia) is further divided into Scleractinia (hard corals, stony corals, true corals, etc.), Zoanthidea (Zoanthids and gold coral), Antipatharia (black corals, whip corals, wire corals, and thorny corals), and Actinaria (sea anemones) orders, while Octocorallia is composed of members of soft corals, gorgonians, sea fans, sea whips, sea feathers, precious corals, pink coral, red coral, golden corals, bamboo corals, leather corals, and horny corals [12,13,14]. Anthozoans are immunologically sophisticated organisms with larger genomes and gene families showing resemblance to those of Bilateria. It is difficult to explain that cnidarians have survived so efficiently with only an innate immune system that acts as a defensive tool against infectious agents. However, these organisms are of great interest because many of their cellular pathways in innate immunity are similar to mammalian immune responses which are absent in other basal invertebrates [15].

Previously, venom was thought to be used mainly for predation in Cnidaria [16,17]. However, cnidarians are now considered one of the two phyla using venom for three prominent ecological functions, which are predation, defense, and intraspecific competition [18]. The extent of the use of venom as a defense tool is highly variable even within closely related groups [19,20]. For instance, in Hexacorallia, nematocyst discharge, when exposed to mechanical and chemical stimuli, was observed in all actiniarian species, with nematocyst secretions in only 40% of zoanthid species and no discharge in the corallimorphians that were tested. These results were reinforced by on-field observations where reef fish showed consistent refusal to consume cnidarians with defensive nematocysts but not to defenseless cnidarians [19]. Another alternative chemical defense strategy in Anthozoa is mediated via poisonous secondary metabolites, particularly in species that dwell in coral reefs [19,20]. In addition, toxin peptides and cells producing nematocysts are distributed across the entire organism, but nematocyte and venom profiles have been seen to vary across morphological structures in species of Actinaria.

A study by Ashwood et al. [21] attempted to understand the relationship between the patterns of toxin expression and the ecological roles of anatomical structures of the sea anemone *Telmatactis stephensoni*. The results revealed that the regionalization of toxin production aligns with partitioning ecological venom functions across envenomating structures, revealing three major functional regions: tentacles, epidermis, and gastrodermis. Structures serving similar functions exhibited comparable putative toxin profiles and nematocyst types. While no overlap existed between toxins identified via proteomics versus transcriptomics, expression patterns of specific milked venom peptides remained conserved across RNA-sequencing and mass spectrometry imaging datasets. Our data suggest that *T. stephensoni* may be transcriptionally inactive, containing only mature nematocysts in distal thread portions. These findings indicate that venom profiles of different anatomical regions in sea anemones vary according to their respective ecological functions [21].

Marine sea anemones exhibit diverse ecological strategies for interspecific interactions and food acquisition, shaped by co-evolutionary processes that encompass mutualistic relationships with clownfish and crustaceans, symbiotic associations with zooxanthellae or zoochlorellae, and predator–prey dynamics involving sea slugs. A study by Durán-Fuentes et al. [22] documented feeding behavior and interspecific interactions of *Actinostella flosculifera*, while characterizing the predatory strategy of the sea slug *Spurilla braziliana* and the corresponding escape mechanism of *A. flosculifera*. The study further revealed that shallow tidal pool habitats (~10 cm depth), occupied by *A. flosculifera*, function as ecological traps, facilitating the prey capture of marine organisms and some biowaste that become stranded during low tide conditions. This is the first documented case of *S. braziliana* predation on *A. flosculifera* and describes interspecific associations between *A. flosculifera* and four crustacean species [22]. These findings collectively demonstrate that sea anemones function as key ecological mediators in marine environments, utilizing specialized venom systems that are anatomically regionalized according to their specific ecological roles—from prey capture and defense to facilitating complex interspecific relationships—and highlight their critical importance as both predators and partners in maintaining the structural and functional integrity of marine community networks.

Calcium carbonate (CaCO_3_) biomineralization represents a 541-million-year evolutionary innovation that has fundamentally shaped species development and global carbon cycling [23]. Within the class Anthozoa, two distinct clades have independently evolved calcification capabilities: Scleractinia (stony corals/Hexacorallia) and Octocorallia (octocorals). Scleractinian corals function as primary reef builders, producing homogeneous aragonite skeletons through well-characterized calcification processes elucidated via skeletal proteomics and immunohistochemistry. Conversely, octocorals exhibit diverse skeletal structures including variable CaCO_3_ polymorphs (aragonite and calcite), organic components such as gorgonin, and distinct sclerite morphologies [24]. This structural diversity in octocorals provides a comparative framework for understanding alternative calcification strategies relative to the established scleractinian model, offering insights into the evolutionary trajectories of biomineralization within cnidarian reef ecosystems. Comparative skeletal proteome analyses revealed that corals with distinguished CaCO_3_ polymorphs utilize a common molecular toolkit that contains cadherin, von Willebrand factor type A, and carbonic anhydrase domains for calcified skeleton deposition. On the other hand, significant divergence exists in collagen distribution, with calcite-forming octocorals exhibiting abundant collagen expression, while aragonitic stony corals show minimal collagen presence. In addition, octocoral collagens have evolved specialized domains associated with matrix adhesion and immune function, potentially representing novel genetic adaptations specific to octocoral calcification mechanisms. These comparative findings illustrate the molecular diversity underlying coral biomineralization strategies and provide foundational insights into octocoral skeletal evolution and formation processes [24]. Venom composition differs significantly across cnidarian classes, where only six proteins out of soluble nematocyst proteins are shared among Scyphozoa, Hydrozoa, and Anthozoa, which mainly have housekeeping functions. The proportion of shared protein content is substantially lower for nematocyst proteins (2%) in comparison to the total proteome (15%) [3]. Venoms of scyphozoans and hydrozoans produce similar biochemical effects; however, sea anemone’s venom is unique as it is dominated by peptide neurotoxins [3,25]. It is hard to determine whether the abundance of neurotoxins is characteristic of anthozoans’ venom unless the taxonomic bias in available data is resolved and knowledge of coral venoms is further elucidated. A comparative analysis of soluble nematocyst proteomes across eight cnidarian species revealed that roughly one-third of identified toxin protein families are shared between Anthozoa and Medusozoa, though Staurozoa was not represented in the study. Among the remaining toxin families, four were restricted to a single taxonomic class, while fifteen were absent from at least one class, with no observable correlation between toxin family distribution patterns and phylogenetic relationships. The apparent loss of multiple toxin families in Cubozoa was linked to a flawed phylogenetic reconstruction that incorrectly positioned Cubozoa as external to both Anthozoa and Medusozoa. This misplacement of Cubozoa likely resulted from the phylogenetic analysis relying solely on the presence/absence of data of known toxins from the ToxProt database rather than more comprehensive molecular data [26].

Actinarians in the subclass Hexacorallia show the highest biological and anatomical diversity [13]. Sea anemones inhabit virtually all marine environments, ranging from deep ocean depths to intertidal coastal areas and from tropical regions to Antarctic waters [13,27], and their widespread distribution lies partly in their capacity to adapt to diverse environmental pressures [28]. In actiniarians, ecological interactions and environmental conditions primarily drive toxin gene expression rather than influence the retention and expansion of toxin gene families themselves [29,30,31]. Comparative studies demonstrate that environmental factors have minimal effects on toxin gene repertoires, showing that phylogenetically related cnidarian species possess more similar toxin gene complements than species sharing the same ecological niche [31]. Additionally, phylogenetic analyses of cnidarian toxin gene sequence variation consistently demonstrate that toxin gene distribution patterns correlate with species evolutionary relationships [32,33,34,35]. These findings indicate that speciation serves as a major driving force in shaping both toxin gene complement and sequence diversity. Nevertheless, ecological factors influencing toxin expression create dynamic spatial and temporal patterns in venom composition [29,30,31,36].

Anthozoans are able to eliminate dangerous microorganisms and also take advantage of associated microbial communities for metabolism, immune defense, development, and behavior [15]. Parisi et al. [15] reviewed knowledge on anthozoan immunity, reporting that they have mechanisms of self-/non-self-recognition, missing adaptive immunity, and discussed signaling pathways and gene transcription activation for defense against pathogens and maintaining homeostasis.

Previous available reviews focused on the phylogenetic relationship of sea anemone toxins, their genes, 3D structures of toxins [25], toxins in corals [37] or cnidarians in general [32,38], cnidarian immunity and defense mechanisms [15], bioactive compounds in zoanthids (sessile colonial anthozoans) with emphasis on alkaloids [39], bioactive metabolites from sea anemones [40], structural overview of sea anemone toxins [41], cytolytic toxins from sea anemones [42], and genomics and transcriptomics of cnidarians, primarily targeting developmental regulatory genes including key bilaterian traits such as mesoderm, nervous system, and bilaterality [43].

In this review, we examined research articles, review articles, and online databases (NCBI) about anthozoan toxins and compounds, chemical defenses, defensive enzymes, and progress in omics for the bioprospection of genomes and transcriptomes in Anthozoa. Our search was performed on Google Scholar, PubMed, and Web of Science, and search parameters were extended to the ‘related articles’ functions.

**Figure 1:**
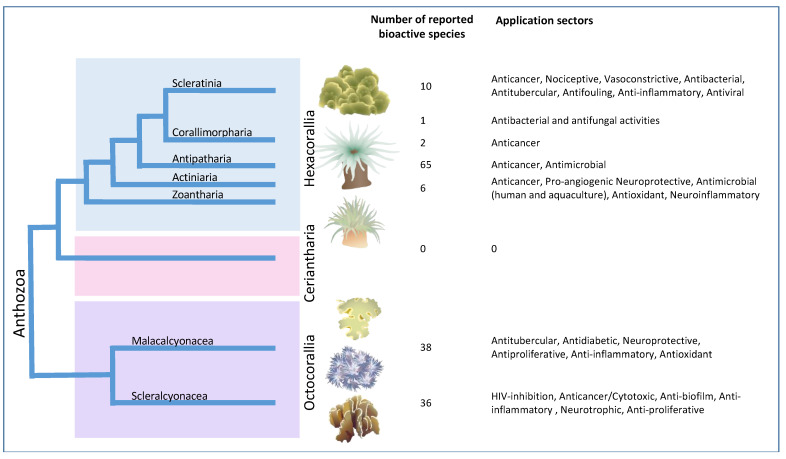
This figure summarizes the number of bioactive species across different orders of Anthozoa, reported in the current review, with the bioactivities of pharmaceutical/biotechnological interests. Attribution for graphical elements: Dieter Tracey, Department of Water, Western Australia; Joanna Woerner; Tracey Saxby, Integration and Application Network (ian.umces.edu/media-library; Accessed on 23 May 2025).

The aim of the current review is to summarize the compounds and toxins identified in different groups of anthozoans and explore their possible pharmaceutical applications (Figure 1). The review further discusses defense strategies in anthozoans with insights into antioxidant enzymes, defensive enzymes in symbionts, and species-specific enzymatic responses. This work also highlights the current state of the art in genomics and transcriptomics of anthozoans, targeting genetic sequences that may be associated with anthozoans’ responses to various stressors, including predation, bleaching events, and climate change.

**Figure 2:**
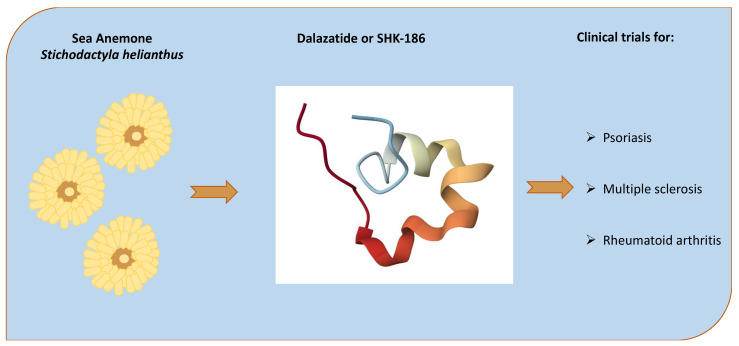
A successful example of a compound from a sea anemone in clinical trials. Protein structure of Dalazatide (SHK-186) was retrieved from PDB, pdb_00004z7p (available under the CC0 1.0 Universal (CC0 1.0) Public Domain Dedication; https://www.rcsb.org/3d-view/4Z7P/1 (Accessed on 4 June 2025).

The efforts to develop a market impact of biomolecules from anthozoans are still in the nascent stages and will need more research and time. However, some anthozoans, for instance, sea anemones, are making progress in the experimental phases and even in clinical trials. A successful example in clinical trials is the peptide ShK, which was first isolated from the sea anemone Stichodactyla helianthus. An analog of this peptide, ShK-186 [44], known as Dalazatide (Figure 2), is currently in Phase 1b-2a clinical trials for treating autoimmune diseases, including multiple sclerosis and rheumatoid arthritis. The selectivity of ShK-186 for voltage-gated potassium channel Kv1.3 over Kv1.1 is reported to be >100-fold greater than ShK [45,46]. ShK-186 has also shown promising results in vitro by modulating CD4^+^T_EM_ cell activity via Kv1.3 blockade and may offer a possible treatment strategy for patients with granulomatosis with polyangiitis (GPA) with high specificity and fewer side effects; Dalazatide is also reported in a Phase 1b trial for the treatment of plaque psoriasis [47,48].

### Defense System in Anthozoans

Cnidarians have no specialized immune cells in their arsenal, yet some cnidarians exhibit specific allorecognition features, for instance, the immunocompetence in colonial hydrozoans and anthozoans characterized by specific reactivity to non-self and succeeding cytotoxic behavior at the colony level, and the presence of a specific memory component in an anthozoan coral (Montipora) [49,50]. Allorecognition is believed to provide protection to colonial cnidarians from mixing with genetically dissimilar individuals and to counteract germline parasitism. These phenomena can be performed with a range of effector mechanisms, including contact avoidance through chemical sensing, use of nematocysts, and barrier formation. For instance, the sea anemone *Anthopleura xanthogrammica* shows tolerance to adjacent coral individuals but also shows aggressive behavior to heterogenic clones with which it may come in contact [51]. Xenorecognition in coral reefs is expressed in the form of distinctive morphological and cytological responses, and colonial reef corals elicit a repertoire of effector mechanisms as a response to xenogeneic contacts [52]. Studies have revealed that single colonies may use simultaneously or separately different effector mechanisms, revealing the capability for the ‘non-self-recognition’ pattern over ‘self-recognition’ [53]. The effector mechanisms in anthozoans during allogeneic or xenogeneic interactions display enormous complexity. The catalog includes chemical sensing to avoid contact, allelopathy, tissue and skeletal outgrowths, barrier formation, developing sweeper tentacles, recruiting mesenterial filaments, forming pseudofusions, retarded growth rates, bleaching, nematocyst firing, onset of delayed responses, necrosis, tissue growth with no calcification, attracting motile phagocytes, and many more (details in Rinkevich [54]. Despite the absence of specialized immune networks, cnidarians, including anthozoans, have evolved well-developed allorecognition and xenorecognition strategies to help them discriminate between the self and non-self, safeguarding colonial integrity via behavioral and chemical adaptations.

In addition to immunity-related responses, anthozoans have evolved to use compounds or toxins as their chemical communication system, defense, or predation tools to ensure their survival in extreme hostile environments. These cnidarians, ranging from pelagic to benthic species, have been shown to be able to produce a repertoire of toxic compounds that may also have antiviral, antibacterial, and anticancer activities [55]. Despite the lack of information about the immune defense system in cnidarians, the tissues and mucus produced by them are involved in defense mechanisms containing a diverse array of peptides, including neurotoxins of sodium and potassium channels, cytolysins, phospholipase A2 (PLA2), and acid-sensing ion channel (ASIC) peptide toxins, among others. These organisms can also benefit from the versatile aspects of some of their toxins; for instance, some bioactive molecules can offer toxicity associated with antimicrobial activity [55]. The interest in investigating anthozoans is not merely to study toxins and venom, but these animals can also be the source of new molecules of considerable interest for biotechnological and pharmaceutical applications.

## 2. Compounds and Toxins

### 2.1. Hexacorallia

Hexacorallia represent a diverse class of anthozoans, and their species diversity is also translated into chemical diversity. This anthozoan sub-class contains all black corals, scleractinians, sea anemones, and tube anemones grouped into several orders (e.g., Actiniaria, Antipatharia, Ceriantharia, Corallimorpharia, Scleractinia, and Zoanthidea). Most Hexacorallians have hexamerous symmetry (as the name suggests), although eight- or ten-part symmetry can also be seen. All species have spriocysts, a type of cnidia with a single-walled capsule and a tubule composed of tiny entangling sub-treads [56].

#### 2.1.1. Zoanthids/Zoantharian

Zoanthids are colonial sea anemones that possess one of the deadliest toxins ever documented, known as palytoxin. It is believed that highly toxic species are not sold commercially for home aquaria; however, the species *Palythoa/Protopalythoa* spp. (*Zoanthus* spp.) were unintentionally introduced into a home aquarium where high concentrations of palytoxin were found, which induced severe respiratory reactions in an individual attempting to remove the contaminated colonies using boiling water [57]. Using genetic analysis of 16S and cytochrome c oxidase (COI), Deeds et al. [58] reported the four zoanthid specimens in three aquarium stores in the Washington D.C. area (*Palythoa heliodiscus*) that were previously responsible for severe respiratory reactions in home aquariums. It was also tested in mice with a lethal dose (LD) of 300 ng/kg [59]; 2 mg of crude toxin from the combined samples can kill 3000,000 mice (standard mouse size of 20 g). Sawelew et al. [60] characterized palytoxin from an undescribed species of *Palythoa* (sister species to *Palythoa aff. clavata*) and found in vitro cytotoxicity (some at picomolar doses) against human cancer cells, including cells from lung carcinoma, glioma, gliosarcoma, and melanoma, making palytoxin among potent anticancer candidates. Palytoxin is also a skin tumor promoter, and apprehending the underlying mechanisms of tumor promotion is mandatory to develop preventive and therapeutic strategies [61]. The ecological function of palytoxin is still controversial, and several hypotheses have been established and debated. The real producer of this toxin appears to be a dinoflagellate; most ecotoxicological studies associated with this compound are focused on the metabolites produced by the genus *Ostreopsis* and not by *Palythoa* [62].

In a study by Chen et al. [63], a novel neuropeptide Y-like polypeptide, ZoaNPY from *Zoanthus sociatus*, was explored for its binding with NPY Y2 receptor (mediating NPY-induced angiogenic response) and proangiogenic activity using an in vitro HUVEC model and an in vivo zebrafish model. Their results revealed that ZoaNPY was able to enhance cell survival, migration, and tube formation in endothelial cells at 1–100 pmol. In addition, ZoaNPY could restore chemically induced vascular insufficiency in zebrafish embryos. The neuropeptide could also enhance the phosphorylation of proteins related to angiogenesis signaling in Western blots. Furthermore, ZoaNPY was able to directly and physically interact with NPY Y2 receptor, showing the pro-angiogenic effects of ZoaNPY involved in activating NPY Y2 receptor, which further activates Akt/mTOR, PLC/PKC, ERK/MEK, and Src-FAK-dependent signaling pathways. These results give possible directions for developing novel pro-angiogenic drugs derived from NPY-like polypeptides in pharmaceuticals [63].

Venoms from marine species have been of interest for mining emerging sources of peptide-based therapeutics, and several peptide toxins from sea anemones have been studied for their pharmacological benefits. Venom complexity can be unlocked with combined approaches of large-scale sequencing and data analysis via integrated transcriptomics and proteomics to annotate new proteins or peptides. Transcriptomic and proteomic analysis of *Zoanthus natalensis* identified six groups of expressed peptide toxins, including neurotoxin, hemostatic and hemorrhagic toxin, protease inhibitors, mixed function enzymes, auxiliary proteins, allergen peptides, and innate immunity-associated peptides. Molecular phylogenetic analysis confirmed the presence and expression of Kunitz-like peptides (similar to Kunitz peptides from snake and spider) in *Z. natalensis* proteome and transcriptome. In vitro bioassays of this peptide, named ZoaKuz1, revealed an intrinsic neuroprotective activity in the zebrafish model of Parkinson’s disease by serving as a voltage-gated potassium (Kv) channel blocker, suggesting a therapeutic role to control neural dysfunction via the inhibition of neurodegeneration triggered by ion-channel hyperactivity [64]. Likewise, three Kunitz-like peptides (PcKuz) were identified in *P. caribaeorum* transcriptome, and in vivo toxicity tests in zebrafish larvae were performed to assess neuroprotective effects. The PcKuz3 isotoxin appeared to be the most neuroactive PcKuz peptide which inhibits 6-hydroxydopamine (6-OHDA) induced-neurotoxicity on locomotive behavior in the zebrafish model and indicates neuroprotective effects of PcKuz3 [65] which may serve as an insightful candidate for treating neurodegenerative diseases.

Marine invertebrates are factories for synthesizing compounds, which have numerous health-improving benefits for humans, and zoanthids are rich in antimicrobial compounds to mitigate some of the problems associated with human pathogens resistant to conventional antibiotics. The antimicrobial potential of the cnidocyst extract from Mediterranean zoanthid coral *Parazoanthus axinellae*, commonly known as yellow cluster anemone, was explored by Stabili et al. [66]. The cnidocyst extract produced remarkable antibacterial activity against human pathogens, such as *Streptococcus agalactiae* (GBS) and *Coccus* sp., against several *Vibrio* species, including microbial agents for humans and aquaculture, mainly, *V. alginolyticus*, *V. anguillarum, V. fischeri, V. harveyi,* and *Vibrio vulnificus* [66]. The antibacterial potential of the *P. axinellae* cnidocyst extract against vibrios, chiefly *V. alginolyticus*, is extremely important for biotechnological applications because the extract might be exploited to combat vibriosis, a significant challenge in aquaculture with heavy economic loss [67,68,69].

In addition, other results have also reported the high antibacterial activity of cnidocyst extract against the bacterial strain *Streptococcus agalactiae* (GBS), a relatively frequent bacterial strain found in the gastrointestinal and genitourinary tract of females. The vertical transmission of the bacteria from mothers to infants at the time of birth is a critical challenge leading to septicemia, meningitis, sepsis, and neonatal pneumonia [66,70,71]. Among newborns, there is a strong relationship between GBS infection and the risk of intrauterine fetal birth [72]. Therefore, finding antibacterial agents capable of fighting GBS is a difficult task due to its high incidence among pregnant women and their neonates, and established antibiotic resistance [73]. The antimicrobial weaponry of *P. axinellae* cnidocyst extract can give new possibilities of fighting infectious agents including GBS bacteria. The *P. axinellae* extract incorporated into nanostructures has demonstrated antimicrobial activity against the Gram-positive strain *Staphylococcus aureus* and Gram-negative strains *Aeromonas hydrophila*, *Aeromonas sobria*, *Escherichia coli,* and *Salmonella enterica* [74]. Different extracts of marine zoanthid *Palythoa caribaeorum* demonstrated antioxidant potential (DPPH radical scavenging assay, ferric-ion chelating assay, and ferric-reducing power), cytotoxicity against *Artemia* nauplii (brine shrimp), and hemolytic activity (to establish whether cytotoxicity is related to damage to the cell membrane or not) against human erythrocytes at 50 µg/mL [75]. These studies suggest that the extracts could have compounds/antioxidants with potential application in pharmaceuticals.

#### 2.1.2. Scleractinia/Stony Corals

Scleractinian corals (stony corals) are the most abundant reef-forming cnidarians found in coral reefs around the world. Most toxicological studies have been performed on Anthozoa; however, the order Scleractinia is poorly explored [76]. Due to their abundance and ecological significance, the knowledge about the diversity of their toxins (bioactive compounds) and their biological functions is important for marine research. Chemical compositions and biological activities of the aqueous extracts of three scleractinian corals collected in the Mexican Caribbean, namely, *Pseudodiploria strigosa*, *Porites astreoides*, and *Siderastrea siderea*, were evaluated for toxicity to crickets (*Acheta domestica*), hemolysis, vasoconstriction, and nociceptive activity. It was reported that extracts were lethal to crickets and induced concentration-dependent hemolytic activity in rat and human erythrocytes due to the presence of cytolysins. Furthermore, the extracts also exerted vasoconstrictor effects on the vascular tone of isolated rat aortic rings and produced significant nociceptive behavior. The presence of phospholipases A2 (PLA2) and serine protease activities was also reported which is responsible for toxicity in scleractinian corals [77]. The isolated aortic rate assay was used to determine whether extracts contained components that induced effects on the cardiovascular system or not [78]. It was further revealed that scleractinian corals can produce low-molecular-weight peptides that can induce toxicity and vasoconstriction [77]. It is noteworthy that cnidarian species can inflict moderate to extreme pain when contacted by humans, and the degree of envenomation depends on the composition of venom and its entry pathway to human skin [79]. The study reported that all extracts produced significant nociceptive behavior during the neurogenic phase. The attempt to obtain extracts of nematocysts from these corals is challenging and justifies the lack of research on the toxicity of these organisms [77].

##### Associated Microbiota

Marine organisms and their associated microbiota are reservoirs of varied chemical compounds of interest in marine biotechnology and have led to a substantial number of research initiatives focused on marine bacteria- and fungi-derived compounds. In this regard, Scleractinia and their associated marine bacteria and fungi are considered at the top of the hierarchy, producing secondary metabolites with promising pharmaceutical applications [80]. About twenty-nine different compounds (eleven xanthones, five sesquiterpenes, four phenyl ethers, five alkaloids, and four other compounds) were detected in the methanol extract of the solid rice culture of fungus *Scopulariopsis* sp., isolated from the inner tissue of coral *Stylophora* collected from the Red Sea in Egypt. The ethyl acetate extract of this fungus on the solid rice medium showed cytotoxicity against the mouse lymphoma cell line L5178Y. Further cytotoxicity investigations disclosed isolated compounds against mouse lymphoma cell line L5178Y which were antibiotic AGI-B4, violaceol I, violaceol II, and scopularide A with IC_50_ (half-maximal inhibitory concentration) values of 1.5, 9.5, 9.2, and 1.2 µM, respectively, compared to the reference drug kahalalide F with an IC_50_ value of 4.3 µM [81]. In another study by Bara et al. [82], the same fungus isolated from the same hard coral near the Egyptian coastline in the Red Sea was studied to explore the metabolic potential when grown on white beans instead of rice media to assess changes in fungal metabolites [82]. As a result of this approach, metabolites were isolated only in this condition, strongly supporting the one strain, many compounds (OSMAC) approach [83]. Two new terpenoids, 3β,7β,15α,24-tetrahydroxyolean-12-ene-11,22-dione and 15α,22β,24-trihydroxyolean-11,13-diene-3-one, along with fourteen known compounds, were reported, including triterpenoids, coumarins, sesquiterpenoids, and polyketides. All the studied compounds were examined for cytotoxic activities against the mouse lymphoma cell line L5178Y, and for antibacterial and antitubercular activities; however, none of them exhibited significant activity, even when the dose was raised to 10 µg/mL [84].

The crude extract of the solid rice culture of marine-derived fungus *Gliomastix* sp., isolated from *Stylophora* sp., contained eight new hydroquinone derivatives, gliomastins A–D, (Gliomastin A), 9-O-methylgliomastin C, acremonin A 1-O-β-D-glucopyranoside, gliomastin E 1-O-β-D-glucopyranoside, and 6′-O-acetyl-isohomoarbutin, together with seven identified analogs. The extract was able to induce cytotoxicity against the mouse lymphoma cell line L5178Y, with the inhibition of 69.1% at a dose of 10 µg/mL [85].

In the case of scleractinian-associated bacteria, a yellowish aerobic marine bacterium, *Erythrobacter flavus* strain KJ5 (formerly called *Erythrobacter* sp. strain KJ5), was found in hard coral *Acropora nasuta* (family: Acroporidae) in Karimunjawa Islands, Indonesia [86], and carotenoids and non-sulphated carotenoids were isolated from this strain. By using an enzymatic assay to investigate the presence of other compounds, the authors showed the discovery of sulfotransferases that catalyze the conversion of carotenoids into carotenoid sulfates, which may be responsible for antithrombotic, antifouling, antiviral, and anti-inflammatory activities [87,88]. A study by Carlson et al. [89] involved a bioassay-guided fractionation to investigate the culture extracts of *Streptomyces* sp. SCSIO 41399, isolated from the scleractinian coral *Porites* sp. (collected from Wenchang, China). This investigation led to the isolation and subsequent identification of eight compounds, which included a novel anthracycline, aranciamycin K, one new tirandamycin analog, isotirandamycin B, and four other anthracycline derivatives. Given the necessity of finding new antibiotics and the preventive role of tirandamycin against vancomycin-resistant *Enterococcus faecalis*, the compounds were tested against *Streptococcus agalactiae*. Compounds such as isotirandamycin B, tirandamycin A, and tirandamycin B were underscored as potent bacteriostatic agents, with minimum inhibitory concentration (MIC) values of 5, 2.5, and 2.5 µg/mL, respectively, compared to erythromycin as a reference drug displaying IC_50_ values of 5 µg/mL [89]. In addition, Scleractinia-associated zooxanthellae have been reported to contain several compounds, for instance, marine sterols isolated from cultured zooxanthellae from coral *Oculina diffusa* [90]. Furla et al. [91] focused their research on the symbiosis between Anthozoa and dinoflagellates, in particular, the dinoflagellate *Symbiodinium* spp., which is also known as zooxanthellae. This symbiosis has influenced species life in different ways: the host has adopted behaviors to optimize the photosynthesis of the dinoflagellates, evolved the ability to absorb and concentrate dissolved inorganic carbon from seawater to supply the algal photosynthesis, developed systems to absorb inorganic nitrogen (which is generally unusual for a metazoan), and has adopted an antioxidant strategy to protect against the oxygen radicals produced during algal photosynthesis. On the contrary, the symbiont produced and transferred to the host sunlight protective molecules, e.g., mycosporine-like amino acids. These findings highlight an example of animal–plant co-evolution.

#### 2.1.3. Sea Anemones/Actinarians

Sea anemones (order Actinaria), also metaphorically known as the flowers of the sea, comprise another important group of Anthozoa, which distinguishes itself from all other cnidarians due to the lack of free-swimming and contains solitary, sessile, and benthic polyps [41,92]. Sea anemone *Actinia equina* is a benthic cnidarian commonly found on the Portuguese rocky shores, formed by a smooth column, usually red, green, or brown, with a blue line on the edge of the base [25]. The components identified are chiefly proteinaceous and are classified as neurotoxins, cytolysins, and Kunitz-type peptides, mediating the process of paralysis, immobilization, and death of the prey [93]. To study the venom system of *A. equina* as a potential source of bioactive compounds with biotechnological opportunities for drug discovery, Alcaide et al. [94] characterized the morpho-anatomy of the venom-shooting apparatus and identified proteinaceous toxins and related bioactive compounds in venom, evaluated toxicity, and compared the venom system between two common morphotypes of *A. equina* (red and green). The toxicity assays revealed that *A. equina* is able to secrete toxins that produce adverse effects on the prey with varied efficiency for each morphotype. Venom extracts of *Actinia* exerted toxicity in zebrafish embryos; green specimen extracts produced a faster toxic effect with lower EC_50_, whereas red specimen extracts caused severe malformations to surviving embryos [95]. The venom’s proteome revealed the presence of proteins of biotechnological interest, toxins being the leading members. A neurotoxin known as Delta-actitoxin-Aeq2a was found in both green- and red-specimen extracts [95], and this protein, also called Ae I, is a type-I sodium-channel inhibitory toxin which, upon binding to voltage-gated sodium channels, delays their inactivation during signal transduction and has exhibited toxicity for crabs and mice [96], suggesting the possible use of toxins (proteins) in biotechnology and biomedicine.

Deep-sea anemones are a rich source of bioactive compounds, and they survive extreme conditions of no light, low oxygen, and high pressure through different biochemical and physiological adaptations that may modify their gene regulation, primary metabolism, and most importantly, the production of secondary metabolites [97,98]. Sea anemones of orders Actiniaria and Corallimorpharia (Coral-like anemones) are prevalent in Oceans, particularly in the Pacific Ocean, and they inhabit the intertidal zones to a depth of over 10 km [99]. A study by Kvetkina et al. [100] identified five species of sea anemones which were collected in the Bering Sea and the Sea of Okhotsk in Russia, and species included *Actinostola callosa*, *Actinostola faeculenta*, *Stomphia coccinea* (family Actinostolidae), *Liponema breviocorne* (family Liponematidae, order Actiniaria), and *Corallimorphus* cf. *pilatus* (order corallimorpharia). The extracts of *Liponema brevicorne* and *Actinostola callosa* displayed the highest hemolytic activity, whereas the extract of *Actinostola faeculenta* showed high cytotoxic activity against murine splenocytes and Ehrlich carcinoma cells. The hemolytic potential of aqueous extracts may be associated with the presence of pore-forming toxins known as actinoporins which may be recruited for health-improving applications in humans.

The extracts of *Corallimorphus* cf. *pilatus* were not toxic to mouse spleen cells; however, the extracts produced the greatest cytotoxic effects against Ehrlich carcinoma cells, most probably due to the presence of neurotoxins or pore-forming toxins. Furthermore, sea anemones *C*. cf. *pilatus* and *Stomphia coccinea* represented promising sources of antimicrobial compounds (antimicrobial peptides) against Gram-positive bacteria (*Bacillus subtilis* and *Staphylococcus aureus*) and antifungal compounds against *Candida albicans*. The aqueous extracts of all sea anemones studied demonstrated α-galactosidase-activating activity which gives an indication of the presence of effectors of this enzyme in the sea anemones [100]. The inhibition of biochemical pathways involving glycosidases via powerful selective inhibitors underscores the treatment of several infectious diseases, malignant neoplasms, and genetic disorders [101], and such an approach can be the cornerstone for searching for novel natural effectors or inhibitors of these enzymes. Deep-sea organisms can, therefore, be treasured for the bioprospection of compounds that might have hemolytic, cytotoxic, antimicrobial, and enzyme-blocking potentials, including anthozoans.

### 2.2. Octocorallia

Octocorals (Cnidaria, Anothzoa, and Octocorallia) are magnificent biorepositories of natural compounds with unique chemical structures and bioactivities which are of the utmost significance to medicine and biotechnology [102]. This subclass contains a wealth of novel, unusual terpenoids. For instance, the diterpenoids caribenol A and B and elisapterosin B isolated from *Pseudopterogorgia elisabethae* (now known as *Antillogorgia elisabethae*), and bipinnapterolide B isolated from *Pseudopterogorgia bipinnata* (also known as Colombian gorgonian Octocoral), are promising compounds possessing antituberculosis potential, inhibiting the growth of *Mycobacterium tuberculosis* in vitro [103,104]. Within Anthozoa, the orders Alcyonacea (soft corals) and Gorgonacea (sea fans) are the ones with the highest number of propitious marine bioactive compounds. The order Alcyonacea contains species that are a potential source of producing secondary metabolites which include diterpenes, sesquiterpenes, furanoditerpenes, terpenoids, capnellenes, and steroids with a diverse range of biological activities (Table 1) that can have a great potential in developing new pharmaceuticals [103].

#### 2.2.1. Soft Corals

Members of the genus *Xenia* (family xeniidae) are rich in diterpenoids; for instance, Xeniolides I, which was isolated from *Xenia novaebrittanniae* (Kenyan soft coral), exhibited antibacterial activity against *Escherichia coli* and *Bacillus subtills* at a concentration of 1.25 µg/mL, while other diterpenoids known as Novaxenicins induced apoptosis in transformed mammalian cells at a similar concentration [238]. A diterpenoid, Blumiolide C, isolated from *Xenia blumi* (Formosan soft coral), demonstrated strong cytotoxicity against mouse lymphocytic leukemia cells (P-388) at a concentration of ED50 = 0.2 µg/mL (Effective Dose 50) and human colon adenocarcinoma cells (HT-29) at a concentration of ED50 = 0.5 µg/mL [237]. Cembranolide diterpene is also a therapeutic anticancer agent from *Lobophytum cristagalli* which has shown the inhibition of farnesyl protein transferase (FPT) with IC_50_ = 0.15 µM [180]. FPT is a crucial protein that mediates signal transduction and the regulation of cell differentiation and proliferation [240]. *Klyxum simplex* synthesizes diterpenes such as Simplexin E, which is found to significantly reduce the level of iNOS (Inducible nitric oxide synthase) and COX-2 (cyclooxygenase-2) proteins in lipopolysaccharide (LPS)-stimulated macrophage cells [178]. *K. simplex* also synthesizes two diterpene compounds, klysimplexins B and H, which show cytotoxic behavior towards human cancer cell lines.

In vitro studies revealed the cytotoxicity of klysimplexins (B and H) towards hepatocellular carcinoma (HepG2 and Hep3B), human breast carcinoma (MDA-MB-231 and MCF-7), human lung carcinoma (A549), and human gingival carcinoma (Ca9-22) cell lines [177]. Other genera of Alcyonacea (soft corals) which contain bioactive arsenal include *Sinularia* (*Sinularia gibberosa*, *Sinularia querciformis*, *Sinularia grandilobata*, *Sinularia flexibilis*, *Clavularia koellikeri*), *Clavularia* viridis, and *Cespitularia hypotentaculata* and exert diverse biological activities such as anti-inflammatory effects, antimicrobial activities, and cytotoxicity, even antifouling properties (e.g., antifouling metabolites), neurotrophic activity, and enzymes inhibiting behaviors [103]. Successively, Eskander et al. [220] studied the chemical defense strategy of the soft coral *Sinularia polydactyla* against biofilm-forming bacteria. They performed different chemical extraction procedures, i.e., by using methanol or hexane as solvent. Both methanol and hexane extracts inhibited the growth of the biofilm-forming bacteria after 4 h of treatment and affected the bacteria’s extracellular polymeric substance production (EPS). They also studied soft coral tissue damage, showing that this had lower antibiofilm activity. Finally, chemical analyses showed that extracts from intact coral were rich in sesquiterpenes, while coral damaged tissues were rich in cembranoids [220]. Ben-Ari et al. [227] studied reef-building corals, showing different nematocyst densities and hemolytic activities. For instance, the coral *Stylophora pistillata*, specifically the tips of the branches, had an increased hemolytic activity compared to the bases. In addition, nematocyst density and hemolytic activity were significantly reduced in species maintained for about one year in captivity compared to the corals sampled from the wild. The authors also identified Δ-Pocilopotoxin-Spi1 (Δ-PCTX-Spi1), a cysteine-containing actinoporin, in *Stylophora* [227]. However, they also noted that, during chromatography, the hemolytic activity was lost, suggesting that other compounds may contribute to that.

#### 2.2.2. Gorgonian Corals

Gorgonian corals (known as sea fans, sea plumes, or sea whips) are prominent members of most tropical and subtropical marine habitats and flourish in the ocean, from the tideland to about 4 km deep in the ocean. These corals are grouped into 13 families comprising more than 6100 species, with the tropical western Atlantic (West Indian) and the Indo-Pacific regions being the two main areas abundant in gorgonian corals. The metabolites produced by these organisms have shown different biological activities such as antioxidant, antiviral, antiplasmodial, antituberculosis, and antitumor activities [241]. Three 8-hydroxybriarane diterpenoids isolated from Gorgonian corals *Junceella juncea*, junceols (A-C), and junceoal A showed the inhibitory effects on superoxide generation by human neutrophils with 45.64%, 159.60%, and 124.14%, respectively [175]. In a study by Chia-Cheng [173], juncin Z (obtained from the Gorgonian coral *Junceella fragilis*) proved to have anti-inflammatory properties by inhibiting the production of superoxide anions by human neutrophils at a concentration of 10 µM. A number of steroid skeletons were discovered in gorgonian coral *Pinnigorgia* sp., which were named as pinnigorgiols (A-E), pinnigorgiol A, compounds containing a rare tricylo [5,2,1,1] decane ring in their structures. Among them, pinnigorgiols D and E were documented to be 11-O-acetyl derivatives of pinnigorgiols A and B, respectively. In vitro investigation concluded that all the newly identified metabolites retained anti-inflammatory potential, with IC_50_ values of pinnigorgiols A-E in the superoxide anion production assay as 4.0, 2.5, 2.7, 3.5, and 3.9 μM, respectively, and inhibitory effects on the release of elastase as IC_50_ 5.3, 3.1, 2.7, 2.1, and 1.6 µM, respectively [197,242]. Another metabolite, known as Apo-9′-fucoxanthinone, isolated from a *Pinnigorgia* sp., displayed inhibitory effects on elastase release by human neutrophils, at a concentration of 5.75 µM [198,199].

In addition to anti-inflammatory properties, sea fans have a repertoire of compounds that induce oxidative stress in cancer cells. Four compounds, including perezone, were obtained from Caribbean gorgonian coral *Pseudoterogorgia rigida* using the bioassay-guided fractionation of the extract. All compounds were cytotoxic towards four human tumor cell lines, perezone being the most cytotoxic but not selective to tumor and non-tumor cell lines. Furthermore, perezone was assayed against HL-60 leukemia cells to examine the mechanisms of cytotoxicity. However, pre-treatment with an acute free radical scavenger (L-NAC) prior to cells’ exposure to perezone eliminated the production of intracellular reactive oxygen species (ROS) and lowered its higher cytotoxicity. These protective effects generated by L-NAC proved that the mechanism of perezone-induced cytotoxicity is partially due to ROS production and a subsequent induction of oxidative stress [211]. Two steroid compounds isolated from the Gorgonian *Isis minorbrachyblasta*, namely 22-epihippuristanol and hippuristanol, have been studied by Qi et al. [172] for their cytotoxicity. Hippuristanol exhibited moderate cytotoxicity against cancer cell lines (A549, HONE1, and HeLa); however, the epimer mixture of 22-epihippuristanol/hippuristanol (3:2 weight ratio) revealed a strong cytotoxic response against A549 and HONE1 cell lines with IC_50_ values of 4.2 and 4.8 µg/mL, indicating a possible synergistic effect on their cytotoxicity against A549 and HONE1 cell lines [172]. Gorgonians and soft corals are also rich in compounds with activity of enzyme inhibition, making these taxonomic groups an ideal target for bioprospecting marine natural products [243].

PTP1B (Protein tyrosine phosphatase 1B) inhibitors have been isolated from numerous species of soft corals including gorgonian corals which can be useful to inhibit PTP1B, known as the target enzyme of new therapeutic drugs (and evaluation of marine compounds as potent inhibitors) in the treatment of type 2 diabetes, obesity, and breast cancer [206]. Novel lipidyl pseudopteranoids, lipidyl pseudopteranes A-F, were isolated from the gorgonian *Pseudopterogorgia acerosa*; among them, lipidyl pseudopteranes A and D demonstrated modest but selective inhibitory activity against PTP1B, suggesting a promising drug target [206]. The enzyme acetylcholinesterase is considered a drug target for the treatment of neurodegenerative disorders, e.g., Alzheimer’s disease [244], for which several inhibitors have been approved by the FDA for clinical use in patients. The most powerful and well-characterized inhibitors to target this enzyme are membranes asperdiol and 14-acetoxycrassine, isolated from the soft coral *Eunicea knighti* and the gorgonian *Pseudoplexaura porosa*, respectively [150].

Similarly, Phospholipases A2 (PLA2) are esterases that cleave off phospholipids and liberate fatty acids and lysophospholipids and are considered promising targets for cancer treatment, as well as inflammation and atherosclerosis [245]. Numerous PLA2 inhibitors have been reported in gorgonian *Euplexaura anastomosans*, described as farnesylhydroquinone glycosides (Euplexides), and Euplexide A, B, and G [152] displayed inhibitory effects against PLA2, from 47 to 71%, at 50 µg/mL in addition to steroid compounds (Table 1) isolated from gorgonian *Acabaria undulata* [106]. IKKbeta is one of the two catalytic units that constitute the kinase complex IkB and is involved in nuclear factor-KB signaling, therefore, in the pathogenesis and progression of inflammatory diseases [246,247]. Folmer et al. [228] were successful in isolating carotenoid astaxanthin from gorgonian *Subergorgia* sp., and it showed inhibitory activity towards the IKKbeta kinase. However, another study revealed that astaxanthin is not synthesized by *Subergorgia* sp., but it is rather acquired from marine bacteria and algae [248], arguing that bioactive compounds can also be produced by bacteria or algae associated with gorgonians. Protein kinase C (PKC) is a family involved in cell signaling pathways that manage important events like cell proliferation and gene expression regulation [249], and this makes PKC a very crucial target for treating several cancers, neurological and cardiovascular disorders [250,251,252]. Gorgonian *Pseudopterogorgia* sp. produces three new 9,11-secosterols with inhibitory effects on PKC at the micromolar scale. Moreover, these three molecules were also evaluated in cell cultures due to the role of PKC in inflammatory and proliferative processes, and the compounds showed antiproliferative potential in cell cultures [154].

A number of 9,10 secosteroids (Astrogorgols A-N) isolated from the gorgonian *Astrogorgia* sp. were assessed against different human tumor-related protein kinases. The kinases evaluated were AKT1 (RAC-alpha serine/threonine-protein kinase), ALK (Anaplastic lymphoma kinase), ARK5 (AMPK-related protein kinase 5), Aurora-B, AXL (AXL receptor tyrosine kinase), FAK (Focal adhesion kinase), IGF-1R (Insulin-like growth factor 1 receptor tyrosine kinase), MEK1wt (MAP kinase 1), METwt (MET receptor Tyrosine Kinase), NEK2 (NIMA-related Kinase 2), NEK6 (NIMA-related Kinase 6), PIM1 (Serine/threonine-protein kinase PIM-1), PLK1 (Serine/threonine-protein kinase PLK1), PRK1 (Serine/threonine-protein kinase N1), SRC (Proto-oncogene tyrosine-protein kinase Src), and VEGF-R2 (VEGFR2 receptor tyrosine kinase). Among the compounds, calicoferol A and E, 24-exomethylenecalicoferol E, 9β-hydroxy-9,10-secosteroid astrogorgol F, and 9α-hydroxy-9,10-secosteroid astrogorgiadiol exerted significant inhibition of kinases LK, AXL, FAK, IGF1-R, METwt, SRC, and VEGFR2 [124]. On the contrary, 9,16 di-oxygenated molecules, including calicoferol I and B, exhibited low inhibition (IC_50_ > 100 µM) against these kinases, indicating oxygenation at C-16 as a cause for suppressed inhibitory activity [124]. The considerable inhibitory potential of 9,10-secosteriods against diverse sets of tumor-associated protein kinases and cytotoxic activities towards tumor cells implied that 9,10-secosteroids [243] may be used as protein kinase targeting inhibitors for the treatment of cancers. In spite of the fact that several enzyme inhibitors were identified in the last five decades from gorgonians and soft corals, not a single one of them possesses complete enzymological characterization. In many of the previous research works, such as that mentioned in a review by Córdova-Isaza et al. [243], only % inhibition at a fixed inhibitor concentration is calculated, and even the IC_50_ value is not described. This trend represents a lack of a uniform procedure for characterizing enzyme inhibitors and users’ unfamiliarity with enzymology [243].

Marine natural products (MNPs), including toxins, are very diverse in marine environments and exhibit a lot of biological activities of pharmacological interest; however, there are challenges associated with the bioavailability and stability of MNPs [253]. For instance, the low bioavailability of marine bioactive peptides (MBPs) represents a limitation due to their susceptibility to degradation by gastrointestinal digestion (of pancreatic, gastric enzymes), membrane enzymes of the small intestine, and under the influence of the acidic environment in the stomach [254,255]. Moreover, the high hygroscopicity of MBPs during storage compromises their stability, resulting in fast microbial and chemical degradation [256]. With recent technologies such as nanoencapsulation and Maillard reaction (MR), there have been promising results in preserving bioactivity, enhancing stability, and controlling the MBPs release with food and nutraceutical applications [253]. Tissue selectivity is a critical concern for any therapeutic agent, where compounds should be cytotoxic against tumor cells and not normal cells. Marine-derived compounds have strong cytotoxic activities against human cancer cell lines; however, they also indicate potential cytotoxicity to normal cells, which limits their therapeutic potential without further structural modification or targeted delivery systems [257], and most of the toxins or compounds in anthozoans display non-selective cytotoxicities.

A major bottleneck to drug development and ensuring sustainability from marine invertebrates is associated with the low biomass of most marine invertebrates, such as wild stocks, low ecosustainability of massive sampling, and, therefore, a very minute amount of pharmacologically active natural products is yielded. The production of pharmacologically active marine natural products can be attained, but in many cases, it lacks economic feasibility due to complex molecular structure and low yields [258]. Taxonomic identification also presents challenges, as many marine organisms cannot be cultured in the laboratory, making them not accessible for taxonomical identification. Such technical criticality can be addressed with recent advanced techniques (e.g., omics) and metagenomics which will alleviate the challenge of identifying and characterizing organisms without cultivation. The criteria for establishing a market demand for marine pharmaceuticals are restricted by long approval times, huge investments from discovery to market, and a high risk of failures due to toxicity and unsustainability [259]. The success of marine-derived drugs will improve with interdisciplinary collaborations, sharing information among stakeholders, collaboration between research institutions and industrial partners, and effective resource management.

## 3. Defensive Enzymes

### 3.1. Antioxidant Enzymes in Anthozoans

Antioxidant enzymes scavenge free radicals to protect cells against destructive oxy-radicals. In the case of increased levels, reactive radicals can result in a far-reaching combination of consequences which include lipid peroxidation, protein degradation, and DNA damage, ultimately inducing tissue damage and cell death [260]. Oxy-radicals have been linked to coral bleaching, and the activities of antioxidant enzymes in host and endosymbiotic algae have been documented. However, to locate the potential cellular targets of oxy-radicals in cnidarians, it is necessary to identify the tissues where these enzymes are active. Some of the antioxidant enzymes for scavenging and neutralizing oxy-radicals include superoxide dismutase/SOD (reduces superoxide to hydrogen peroxide (H_2_O_2_) and O_2_), catalase/CAT (converts H_2_O_2_ to 2H_2_O and O_2_), and glutathione peroxidase/GPX (converts reduced glutathione to oxidized glutathione and water) [261].

The antioxidant enzymes SOD, CAT, and GPX were localized in temperate sea anemone *Anemonia viridis* and tropical coral *Goniopora stokesi* by using immunocytochemical techniques. Further investigations involving the use of affinity-purified primary antibodies and transmission electron microscopy (TEM) showed that antioxidant enzymes were associated with granulated vesicles, accumulation bodies of endosymbiotic algae, and cnida. SOD and CAT gold-labeling were found in all forms of cnida; SOD was predominantly found on ruptured threads and shafts on b-mastigophore in *A. virdis*, likely suggesting that the b-mastigophore had endured autolysis and needed SOD to prevent damage to host cells. The presence of SOD and CAT in the accumulation body of endosymbiotic algae agrees with the presupposed role of these bodies in digestion and cell aging. CAT was also localized in isolated electron-rich bodies, often adjacent to microvillous borders in *G. stokesi*. Similar bodies were documented in *A. viridis* but composed of GPX instead of CAT, and GPX was also found in symbiotic algae, where it was associated with electron-rich bodies [261].

Corals are known for their fascinating appearance (coloration), largely due to fluorescent proteins (FPs). FPs are abundant and diverse in anthozoans with four basic color types: red (REP), green (GFP), cyan (CFP), and blue/purple non-fluorescent chromoprotein. However, their biological function in a symbiotic association is less understood and controversial. In a study, the presence of FPs was determined and quantified for seven Caribbean hard coral species (*Montastraea annularis*, *Montastraea faveolata*, *Montastraea cavernosa*, *Diploria strigosa*, *Porites astreoides*, *Dichocoenia stokseii*, and *Sidastrea siderea*) aided by the spectral emission analysis of tissue extracts. There was a positive correlation between FP concentration and H_2_O_2_ scavenging rates both in vivo (across multiple species) and in vitro (with purified proteins), showing antioxidant potential of tissue extracts [262]. Clarke et al. [263] demonstrated the antioxidant capacity of AnthoYFPs against oxidative stress of H_2_O_2_, UV light exposure, and thermal shock (37 °C), a subfamily of GFPs obtained from three species of intertidal sea anemones (*Anthopleura elegantissima*, *Anthopleura sola*, and *Anthopleura xanthogrammica*). The results revealed a higher frequency of dead cells in YFP-negative cells than in YFP-positive cells, with the strongest effects observed in H_2_O_2_ treatment. A similar effect was achieved by treatment with a different oxidizing agent, tert-butyl hydroperoxide, suggesting that AnthoYFP exerts an impartial protective effect, no matter what the source of reactive oxygen species (ROS) may be [263].

### 3.2. Enzymes in Symbionts

Most corals and sea anemones live in symbiosis with photosynthetic microorganisms (microalgae) known as zooxanthellae. The photosynthetic process of endosymbionts produces a hyperoxic state, which requires an efficient defense strategy in host cells against ROS [264,265]. To address this challenge, symbiotic cnidarians recruit diverse array of SOD isoforms, for instance, copper- and zinc-containing SODs (CuZnSODs) in a diploblastic organism such as anthozoan sea anemone *Anemonia viridis* [266]. To understand the mechanism of resistance of anthozoan hosts to hyperoxia, two CuZnSOD genes (named as AvCuZnSODa and AvCuZnSODb) were cloned into pGEM-Teasy vector (Promega), and molecular analysis revealed that the AvCuZnSODa transcript encodes an extracellular form of CuZnSOD, whereas the AvCuZnSODb transcript encodes an intracellular form. Upon in situ hybridization, both gene transcripts were documented to be expressed in endodermal and ectodermal cells of the sea anemone *Anemonia viridis*, not in zooxanthellae [267], representing a perfect example of effective defensive tools in the host when presented with harmful signals from endosymbionts. Proteins exhibiting resistance to hypoxia may be used for developing SOD mimics of biomedical and cosmetic interests [268,269]. Higuchi et al. [270] studied activities of SOD and CAT in a colony of corals *Galaxea fascicularis* with elevated concentrations of H_2_O_2_ in seawater using incubation chamber, and compared changes in enzyme activity to those induced by increased seawater temperature. It was found that CAT activities (in coral tissue and zooxanthellae) increased with elevated H_2_O_2_, but SOD activity remained relatively constant, suggesting that the spike of H_2_O_2_ in seawater affected coral cytol but did not trigger superoxide formation. On the contrary, increased seawater temperature led to elevation in both SOD and CAT activities in coral tissue and zooxanthellae during short term exposure (5-day period). This may be inferred that coral bleaching would likely not happen from short-term exposure to H_2_O_2_ concentrations in seawater [270].

Ramos and Garcia [271] investigated the cytochrome P450 monooxygenase (MFO) system and antioxidant enzyme responses in the scleractinian coral *Montastraea faveolata* when exposed to the organic contaminant, benzo(a)pyrene (B(a)P). Corals were subjected to 0.01 and 0.1 ppm B(a)P concentrations for 24 and 72 h, with enzymatic activities measured in host (polyp) and symbiotic zooxanthellae cells. Antioxidant enzymes catalase (CAT), superoxide dismutase (SOD), and glutathione S-transferase (GST) showed significant increases at the highest concentration and prolonged exposure time. Cytochrome P420 was present in all colonies, while cytochrome P450 content peaked in colonies exposed to the highest contaminant concentrations. NADPH cytochrome *c* reductase activity and pigment concentrations remained consistent across treatments. This research represents the first documented evidence of a detoxification mechanism induced in *M. faveolata* during acute organic contaminant exposure, highlighting the coral’s physiological response to environmental pollutants [271]. This study also acknowledges the induction of biotransformation and antioxidant enzymes in corals exposed to organic contaminants. Among other environmental stresses, Liñán-Cabello et al. [272] studied the short-term exposure of reef-building coral *Pocillopora capitata* to photosynthetically active radiation (PAR) and ultraviolet radiation (UVR) over 32 h. Exposure to UVR resulted in lower carotenoid levels and antioxidant enzyme (SOD, CAT, GPx, and GST) activities compared to PAR, with reduced carotenoid-pigment-to-chlorophyll ratio. Despite rapid production of non-enzymatic antioxidants like mycosporine-like amino acids (MAAs) and carotenoid pigments, these mechanisms were insufficient to prevent reactive oxygen species (ROS) damage, leading to zooxanthellae expulsion at 33 times more than the rate observed in PAR treatments. The coral demonstrated short-term enzymatic adaptations to resist ROS propagation, potentially enabling survival in high UVR environments. However, additional environmental variables like turbidity, sediment, nutrients, temperature, and osmolarity could interact to cause irreversible damage, underscoring the need for a comprehensive management plan for Mexican Pacific coral reefs [272].

### 3.3. Species-Specific Enzymatic Response

Different species of corals exhibit species-specific susceptibility and tolerance under the same conditions. A study in 2021 examined shipping-induced stress in two octocoral species, *Sinularia polydactyla* and *Sinularia asterolobata*, transported from Indonesia to Europe, by assessing oxidative stress markers, energy reserves, and cellular damage upon arrival and after three months. *S. polydactyla* showed immediate detoxification efforts through increase in the second line of oxidative defense (increase in glutathione S-transferase (GST), total glutathione (tGSH) activities, and depleted CAT), but ultimately perished within 24 h of arrival. *S. asterolobata* activated antioxidative pathways (GST, CAT, and tGSH) post-shipping and demonstrated long-term adaptability, though experiencing significantly elevated lipid peroxidation levels after three months. The research underscores species-specific responses to shipping stress and the critical need for tailored transportation strategies to minimize biomass loss in coral trade and research contexts [273]. In Spain, the snakelocks anemone (*Anemonia viridis*) is a highly valued seafood product; Coll et al. [274] evaluated the physiological response of the snakelock anemone to different biotic and abiotic factors in an aquaculture system by assessing oxidative defense enzymes in tentacular and columnar tissues. The study evaluated multiple antioxidant enzymes including SOD, CAT, GPx, glutathione reductase (GR), glucose 6-phosphate dehydrogenase (G6PDH), glutathione S-transferase (GST), and DT-diaphorase, along with Trolox-equivalent antioxidant capacity (TEAC) and Malondialdehyde (MDA) for lipid peroxidation. Brackish water and integrated multitrophic aquaculture (IMTA) conditions triggered significant changes in glutathione-related enzymatic pathways, particularly in columnar tissue, while reduced light exposure did not compromise the species’ oxidative status despite its symbiotic relationship with photosynthetic organisms. Such studies stressed the improvement in environmental conditions in aquaculture and also the importance of the enzymatic machinery in anthozoan species [274].

The investigation of immune responses in two Argentinian sea anemone species, *Aulactinia marplatensis* and *Bunodosoma zamponii*, by examining phenoloxidase and peroxidase activities across their ectoderm, endoderm, and tentacles. While both enzymes were detected throughout all tissues, *B. zamponii* demonstrated notably higher phenoloxidase production, suggesting enhanced disease resistance and stress tolerance compared to *A. marplatensis* [275]. Palmer et al. [276] revealed three melanin-synthesis pathway components, mono-phenoloxidase, ortho-diphenoloxidase (tyrosinase-type pathway), and para-diphenoloxidase (laccase-type pathway), in their active form known as phenoloxidase (PO) and inactive form known as prophenoloxidase (PPO), in 22 diverse species of Indo-Pacific anthozoans, including 18 hard corals, 3 soft corals, and a zoanthid. Melanin synthesis enzymatic activities varied among taxa, and inactive tyrosinase-type activity (PPO) and active laccase-type activity (PO) correlated with taxonomic patterns in diseases resistance, whereas the negative relationship between bleaching susceptibility and stored enzymes of less cytotoxic laccase pathways at the family level suggested that melanin production from this pathway may increase bleaching resistance, possibly via protection against light-induced damage without extreme cytotoxicity of the tyrosinase-type pathway [276].

To investigate specific inflammatory responses of *Anemonia sulcata* when exposed to pathogenic threats, Trapani et al. [277] studied enzymatic activity following bacterial injections of *Escherichia coli* and *Vibrio alginolyticus*. By analyzing the enzymatic activity of protease, phosphatase, and esterase, it was revealed that the injection of different bacterial strains alters the expression of these enzymes, implying a correlation between the appearance of an inflammatory reaction and modification of enzymatic activities. Apart from enzymes, enzyme inhibitors have been isolated from sea anemones. For instance, a peptide inhibitor of mammalian α-amylases, Magnificamide, has been isolated from the sea anemone *Heteractis magnidica*, which has the potential application in controlling postprandial hyperglycemia in diabetes mellitus [278] by inhibiting α-amylases and resolving the challenges of high immunogenicity associated with bacterial-derived polypeptide inhibitors.

## 4. Molecular Resources Available: Genomes and Transcriptomes

The appearance of the genomic era has stipulated crucial and astonishing insights into the genetic composition of the common ancestor of cnidarians and bilaterians. This has advanced our understanding of how metazoan genomes evolved and when important gene families arose and diverged in animal evolution. By sequencing several cnidarians’ genomes, it has been revealed that cnidarians have a great repertoire of genes which show genome synteny with vertebrates, with fewer gene losses in the anthozoan cnidarian lineage than ecdysozoans (*Drosophila melanogaster* or *Caenorhabditis elegans*) [43]. Cnidarian genomes also possess a rich repertoire of transcription factors, including those that in bilaterian model organisms regulate the development of key bilaterian traits, for instance, mesoderm, nervous system, and bilaterality. Overall, the genomics and transcriptomics analysis in anthozoan cnidarians suggest that the most conserved genes in our genomes and mechanisms guiding their expression have evolved prior to the divergence of cnidarians and bilaterians about millions of years ago [43].

Looking on the public database GenBank/NBCI (https://www.ncbi.nlm.nih.gov/genbank/; accessed on 7 April 2025) and searching for anthozoans, a total of 282 genomes were found. However, the genomes, which were annotated either by NCBI RefSeq or by the GenBank submitter, were considered. The search revealed that there are 24 genomes (or 28, where some of the genomes are annotated by both methods) available for Anthozoa. From the NCBI database, it was possible to retrieve significant features such as assembly accession number, organism name, assembly release date, sequencing technology, total genes (annotated), and protein-coding genes (annotated) (as reported in Table 2).

Several authors also combined genomic and transcriptomic responses in anthozoans in order to better understand the species’ response to specific abiotic and biotic stressors. Some examples of habitats/conditions associated with genetic pathways are described in the sections below.

### 4.1. Genomes and Transcriptomes in the Deep Sea

Deep-sea hydrothermal vents and cold seeps are characterized by darkness, extreme hydrostatic pressure, and the presence of reducing chemicals such as hydrogen sulfide and methane that serve as energy sources to fuel chemosynthesis. They represent a different ecological niche for those organisms that depend on photosynthesis for production and, as such, provide us with very distinctive ecological and evolutionary systems from those commonly studied. The genome (including 30164 protein-coding genes and 14806 tRNA genes) of deep-sea anemone *Actinernus* sp. was reported to contain a mega-array of ANTP-class homeobox genes. The analysis of homeobox genes disclosed that the longest chromosome hosts a diverse array of Hox clusters, Hox-linked (HoxL) homeobox genes, NK clusters, and NK-linked (NKL) homeobox genes, and the presence of these genes may suggest an ancient ancestral state for these key developmental control genes responsible for molecular adaptations to deep-sea habitats [279]. In addition, the poorly understood tissue (tentacle) regeneration in cnidarians due to non-coding RNAs such as microRNAs (miRNAs) has been investigated using transcriptomics [280]. The sequencing and assembling genome of the sea anemone, *Exaiptasia pallida*, were carried out after tentacular excision at nine time points, from 0 h to 8 days. The study demonstrated that, in addition to Wnt signaling pathway and ANTP-class of homeobox genes that are previously reported to be involved in tissue regeneration in other cnidarians, GLWamide neuropeptide (out of 4 annotated neuropeptides) and sesquiterpenoid pathways genes may contribute to the late phase of cnidarian tissue regeneration. The expression profiles of genes involved in sesquiterpenoid biosynthetic pathways indicated the down-regulation of Acetyl-CoA acetyltransferase (ACAT) and the isoprenylation pathway genes. During the nine time points, 127 mRNAs and 141 miRNAs were up-regulated, and 58 mRNAs and 4 miRNAs were down-regulated [280].

The adaptations in sea anemones to hydrothermal vents have been studied through the lens of comparative transcriptomics of deep-sea anemones and shallow water sea anemones. Xu et al. [281] sequenced the transcriptome of the hydrothermal vent sea anemone *Alvinactis* sp. to elucidate the mechanism of adaptation to vent conditions and compared it to another deep-sea anemone (*Paraphelliactis xishaensis*) and five shallow water sea anemones. There was a total of 117 positively selected genes and 46 significantly expanded gene families reported in *Alvinactis* sp., which may contribute to vent-specific adaptations, providing the first transcriptome of sea anemones that are inhabitants of hydrothermal vents and an extreme environment. Sea anemone venom is a marine drug resource, not only for the defense system in the organism, but also has value in pharmacology and biotechnology. In a study by Fu and colleagues [282], a transcriptomic approach was used to sequence venom components of different developmental stages of the sea anemone *Exaiptasia diaphana*, and 533 putative proteins, as well as peptide toxin sequences, were found. The 533 identified transcripts were classified into 75 known superfamilies based on predicted functions, 72.98% proteins and 27.02% peptides. Protein constituents primarily corresponded to metalloproteases, chymotrypsinogen-like, pancreatic lipase-related protein-like (PLRP-like), G-protein-coupled receptor and collagen, while peptide sequences correspond to the ShK domain, thrombin, Kunitz-type, and insulin-like peptide and defensin [282].

Other sequencing projects on early-diverging metazoans such as cnidarians have focused on the innate immunity gene repertoire. An example is the study by Goldstone [283] in 2008 which, by using a genome and transcriptome search, looked for sequences coding proteins involved in the chemical defensome of the starlet sea anemone *Nematostella vectensis*. The study showed the presence of several sequences related to receptors and signal transduction, efflux transporter proteins, oxidative, reductive, and conjugative biotransformation enzymes, as well as antioxidants, heat shock proteins, and metal detoxification enzymes. Interestingly, the absence of specific proteins was reported, for example, the absence of metallothionein genes, suggesting gene loss. With this comparative analysis, the presence of 266 genes belonging to the sea anemone defensome was reported, compared to 218 in humans, 270 in tunicates, and 423 in sea urchins [283].

There is little information about immunity-associated gene regulation in the host’s early response against bacterial infections in marine environments. Seneca et al. [284] used RNA-seq symbiotic sea anemone *Exaiptasia pallida* strain CC7 as a model species to illustrate innate immune response to *Vibrio parahaemolyticus* strain infection and lipopolysaccharides (a Gram-negative specific endotoxin) exposure. Analysis focused on three main objectives: genes differentially expressed in infected anemones, gene expression variation over the onset of infection, and comparison between responses in both types of exposures. Gene expression and functional analysis documented hundreds to thousands of genes responsive to bacterial infection at different exposure periods. The results indicated that non-canonical cytoplasmic pattern recognition receptors (PRRs) such as NOD-like and RIG-I-like receptor homologs take part in the molecular immune response in *E. pallida*. Moreover, several members of lectin-complement pathways were over-expressed in parallel with novel transmembrane and Ig (immunoglobulin) containing ficolins (CniFLs), suggesting a potent defense against pathogens. Interestingly, the sea anemone lacked typical Toll-like receptors (TLRs), while a TLR-like pathway, including up-regulated MyD88, TRAF6, NF-κB, and AP-1 genes, was activated in the organisms, which were not induced by lipopolysaccharide exposure, proposing an alternative ligand-to-PRR activation. The study further revealed the activation of cytokine-dependent signaling pathways as part of the innate immune response following vibrio exposure, two of which (involving tumor necrosis factor rectors (TNFRs) and several downstream signaling genes) could induce an inflammatory response and/or apoptosis [284]. Venom in the sea anemones that have co-evolved with clownfish via a comparison of transcriptomes was studied in a clownfish-hosting anemone representing each of three major clades of sea anemone hosts: *Entacmaea*, *Stichodactylina*, and *Heteractina*. By investigating transcriptomic data to identify key differences and similarities in venom profiles, in 1121 transcript-matching-verified toxins across all species, hemolytic and hemorrhagic toxins were most dominant [285].

Genomic efforts towards corals have been substantially extended in recent years; genomic and transcriptomic data now in existence for at least 20 coral species, along with comparative molecular studies in corals, have identified genes responsible for biomineralization, symbiosis, and environmental responses [286] and can help us understand the evolution of specific immune gene repertoires in corals. The genome of scleractinian coral *Pocillopora damicornis* was sequenced and annotated by Cunning et al. [287] to find answers to three critical questions about (1) genes that are either unique to or show diversification within the Scleractinia lineage, (2) genes that are specific to or diversified within individual scleractinian coral species, and (3) features that distinguish the *P. damicornis* genome from other corals’ genomes. The results reported that 46.6% of genes had orthologs in all other scleractinians, signifying the presence of basic housekeeping genes in the coral’s core genome. Among these core genes, 3.7% were specific to scleractinians with immune functionality, which may translate into an important role in immune processes in coral evolution. Genes only unique to *P. damicornis* were enriched in cellular signaling and stress response pathways, and such immune-associated gene family expansions were found in each coral species, which emphasizes immune system diversification at various taxonomic levels. Most of the *P. damicornis*-specific genes were unannotatable in the study; however, protein domain homology disclosed significant enrichment for 11 GO terms, which included the GPCR signaling pathway, bioluminescence, activation of NF-κB inducing kinase, and positively regulated Jun N-terminal cascade (JNK) cascade [287].

### 4.2. Genomes and Transcriptomes in Response to Abiotic and Biotic Stressors

Climate change factors such as increased sea surface temperatures and ocean acidification can disrupt the symbiotic relationship between reef-building corals and their algal symbionts in the event of coral bleaching. Coral bleaching further increases the chances for disease outbreaks which can permanently modify reef ecosystems. Whole (meta)transcriptome analysis was used by Pinzón et al. [288] to assess the effects of a natural bleaching event on genes involved in the innate immune system of Caribbean coral *Orbicella faveolata*. The findings revealed that each portion of the holobiont (*O. faveolate*, algal symbiont *symbiodinium* spp., and other eukaryotes (e.g., endolithic algae, fungi, ciliates, etc.)) has distinguished responses to bleaching and recovery from bleaching, where the coral host response appeared to be masked by responses of the associated organisms. Coral bleaching changed the expression of genes associated with innate immunity, and these effects lasted (at least one year), even after the recovery of symbiotic populations [288]. In addition to vulnerability to thermal stress in the extensive regime of climate change, *O. faveolate* is also a victim of disease outbreaks in marine ecosystems. Colonies of *O. faveolata* were exposed to lipopolysaccharides (LPS), bacterial pathogen-associated molecular patterns (PAMPs), and changes in profiles of gene expression and protein activity were examined by Fuess et al. [289]. Differential expression analysis identified 17 immune-related transcripts that could be classified into one of the three processes of immunity: recognition, signaling, and effector response. Network analyses demonstrated several groups of transcripts correlated to immune protein activity; several transcripts annotated as positive regulators of the immunity were included in these groups, and some were down-regulated after LPS exposure [289]. The reported pattern of dysfunctional gene expression and protein behavior may explicate the processes responsible for disease susceptibility in coral species.

The effects of thermal stress on coral immunity against *Vibrio coralliilyticus* were investigated through whole-genome transcriptomic analysis in the primary polyp of the Coral *Acropora digitifera*. The authors reported that bacterial invasion suppressed gene expression concerning innate immune response, mainly down-regulating toll-like receptors (TLRs), nucleotide-binding oligomerization domain-containing proteins (NODs), myeloid differentiation primary response protein (MYD88), and NOD-like receptors (NLRs) under thermal stress. Additionally, to neutralize the infected pathogens, the coral employed complex changes such as altered mitochondrial metabolism and protein metabolism, exosomal intercellular communication for delivery of biochemical cues (e.g., microRNA, proteins, and lipids), and extracellular matrix (ECM) remodeling [290]. In another study by Libro et al. [291], next-generation RNA-seq was used to generate a transcriptome-wide profile of the immune response of the Staghorn coral *Acropora cervicornis* to White Band Disease (WBD) by comparing healthy (asymptomatic) and infected coral tissues. Differentially expressed transcripts were documented in coral and non-coral datasets to identify gene sequences that are up- and down-regulated due to infection. Their findings revealed that infected coral exhibited substantial changes in gene expression across 4% of coral transcriptome, and the transcripts (of infected coral) were involved in responses such as macrophage-mediated pathogen recognition and ROS production, phagocytosis (Macrophage receptor multiple epidermal growth factor-like domains protein 10 (MEGF10) and actin-22 (act22)) and key mediators of apoptosis (up-regulated tumor necrosis factor receptor superfamily member 1A (TNFRSF1A) and caspase 3 (CASP-3), and up-regulated calcium homeostasis. Furthermore, an enzyme known as allene oxide synthase-lipoxygenase was also up-regulated, suggesting its role in allene oxide pathways in coral immunity. Surprisingly, none of the three primary innate immune pathways—Toll-like receptors (TLRs), Complement and prophenoloxydase pathways—were strongly involved with response of *A. cervicornis* to infection, and the 52 putative *Symbiodinium* or algal transcripts had no contribution to coral functions, proposing that immune response is mediated by coral host, not by its symbiont [291].

### 4.3. Genomes and Transcriptomes to Study Toxins and Bleaching Events

The transcriptome of zoanthid *Protopalythoa variabilis* was investigated for the presence of peptide toxin-related components in its tissues, and several predicted polypeptides with canonical venom protein features were identified. These polypeptides consist of putative proteins belonging to diverse toxin families, including neurotoxic peptides, hemostatic and hemorrhagic toxins, membrane-active (pore-forming) proteins, protease inhibitors, mixed-function venom enzymes, and venom auxiliary proteins. The functional analysis of two predicted toxin products, Shk/Aurelin-Like Peptide and Anthozoan neurotoxin-like peptide, demonstrated in vivo neurotoxicity that impaired swimming in larval zebrafish. The complex array of venom-related transcripts that are identified in *P. variabilis* provides insight into toxin distribution among soft corals and can help in comprehending the evolution of venom polypeptides in toxiferous organisms [292]. Diterpenes are major defensive small molecules that help soft corals to survive without a tough exterior skeleton. The discovery of coral defensive biosynthetic genes would prove that marine animals, in addition to their symbionts, can produce defensive molecules, which pinpoint a key biochemical event that has emerged in the soft-bodied corals and also provide information for bioprospecting marine drugs. Scesa et al. [293] described the discovery of terpene biosynthetic gene clusters (BGCs) by using genomic and transcriptomic sequencing of eleutherobin producer *Erythropodium caribaeorum*, which led to heterologous expression and in vitro characterization of two diterpene-producing enzymes. These coral-encoded terpene cyclase genes synthesize the eunicellane precursor of eleutherobin and cembrene, representative precursors for more than 2500 terpenes found in octocorals, implying that these terpene cyclases mediate an ancient evolutionary role in coral defense.

The blue coral *Heliopora coerulea* inhabits shallow water habitats and demonstrates the optimal growth rate at a temperature which is very close to the temperature threshold causing bleaching in scleractinian corals. Guzman et al. [294] attempted to understand the molecular mechanisms involved in the biology and ecology of *H. coerulea* by generating a reference genome of this coral by next-generation sequencing. Metatranscriptome assembly contained transcript sequences from both the coral host and its symbiont, a thermotolerant C3-Gulf ITS2 type *Symbiodinium*. The transcriptome of the blue coral displayed several gene families involved in stress response, including heat shock proteins and antioxidants that may be associated with maintaining cellular homeostasis, and genes associated with signal transduction and stimulus response [294]. The sea fan coral, *Gorgonia ventalina*, has suffered large-scale declines in the Caribbean in the 1990s due to an infection known as Aspergillosis caused by a fungal pathogen *Aspergillus sydowii*. A sea fan pathogen, an *Aplanochytrium* spp., which is a marine stramenopile protist also damaged the host, mainly through the longitudinal tearing of the host gorgonin (skeleton) and degradation of the host’s polyps. Burge et al. [295] used short-read sequencing (Illumina GAIIx) to generate a transcriptome of the sea fan coral and to characterize the sea fan host response to *Aplanochytrium* spp. using RNA-seq analysis. The analysis revealed the presence of 210 differentially expressed genes (DEGs) in sea fans exposed to the *Aplanochytrium* parasite. Several DEGs had putative immune functions such as the role in pathogen recognition (e.g., Tachylectin-5A, Protein G7c, and Neuronal pentraxin-2), genes involved in wound healing (Matrix metalloproteinase or peroxidasin), and antimicrobial peptides (e.g., arenicin-2 and royalisin). Functional enrichment analysis identified that the majority of enriched genes encoded ribosomal proteins involved in protein translation and energy production, and all these genes were up-regulated in exposed sea fans [295].

Finally, Shinzato et al. [296] focused on a genome analysis approach to investigate the defense system of the coral *Acropora digitifera*. The identified genes included some xenobiotic receptors, transcription factors, antioxidants, metal-related enzymes, and heat shock proteins. Also, in this case, the authors did not find any metallothionein, but found various multicopper oxidases and one phytochelatin synthase [296] Such research on the key aspects of sea fan immunology will enable further studies targeting environmental drivers of disease and host immunity, and also reveal important genes in invertebrate innate immune pathways. These efforts to explore the genomes or transcriptomes of different classes of anthozoans provide a broad understanding of the mechanisms of resistance, evolution, or adaptation in marine organisms in response to different stresses, including defense against predation, infections, and bleaching events.

## 5. Conclusions

A number of different compounds and toxins were identified in diverse members of the subphylum Anthozoa, and their biological activities, along with their structures (when available/possible), were reported in the current review. The species diversity in Anthozoa also translates into chemical diversity, which makes it quite challenging for cross-comparison across scientific studies. In addition to the richness of toxins/compounds, the diversity of the research methodologies used also makes it difficult to perform comparisons between anthozoan studies.

Some of the compounds and toxins showed bioactivities as potential drug candidates which can improve the current state of pharmaceuticals for human treatments. These compounds were produced in response to the danger of predations, climate change (e.g., thermal stress), and as part of symbiotic associations, which represent the defensive strategies that have evolved over a period of millions of years. Among them, numerous compounds have demonstrated biological activities which are useful for human health, for instance, inhibiting microbial growth, mediating inflammations, producing anticancer or antiproliferative effects against an array of cancer cell lines, and HIV-inhibitory activity. Additionally, some of the compounds or their extracts can help in alleviating the multi-drug resistance not only in humans but also can be used in biotechnological applications in aquaculture. Anthozoan molecules were able to alter various cascade pathways in human cells, such as those related to NF-κB and Jun N-terminal cascade (JNK), and considering that these pathways are known to be involved in various inflammatory and cancerous pathologies, they represent a valuable source for new drug candidates. The active concentrations of the compounds and/or extracts ranged from an MIC value of 2.5 µg/mL (tirandamycin A and tirandamycin B) to 5 µg/mL (sotirandamycin) as potential bacteriostatic agents, cytotoxicity of Blumiolide C ranged from an ED value of 0.2 µg/mL (against mouse lymphocytic leukemia cells (P-388)) to 0.5 µg/mL (against human colon adenocarcinoma cells (HT-29)), and the anti-inflammatory potency of junicin Z at a concentration of 10 µM by inhabiting superoxide anions produced by human neutrophils to promising results of Apo-9′-fucoxanthinone at 5.75 µM of inhibitory effects on elastase secretion by human neutrophils. Experiments have been performed in vitro and in vivo using models such as zebrafish and brine shrimps, which further strengthens the promising results of using anthozoan-derived compounds for possible future drugs from the sea.

This review provides an overview of known chemical defense strategies within Anthozoa and acknowledges the limitations in taxonomic coverage due to the group’s vast diversity and the uneven availability of biochemical data. Several lineages remain underexplored, and their potential for unique defensive metabolites is yet to be uncovered. Future research should prioritize broadening taxonomic sampling and integrating chemical, ecological, and phylogenetic approaches to fully understand the diversity and evolution of chemical defenses across Anthozoa.

## Figures and Tables

**Table 1:** This table reports compounds/toxins found in Anthozoans, their reported activities, and related references. Abbreviations used: ROS stands for reactive oxygen species; NO for nitric oxide; BV-2 for murine microglial cell line; HCT-8,MDA-MB-435, SF-295, HL-60 for different human cancer cell lines; U251 and SKLU-1 for two cancer cell lines; RBC for red blood cell; HepG2 and MCF-7 for cancer cell lines; WI 38 and VERO for normal (non-cancerous) cell lines; L-929 for normal cell line; HeLa for a cancer cell line; Tax for Tax protein; CREB for cAMP response element-binding protein; Myc for c-Myc protein; Max for Myc associating X-protein; Mic-1 for Macrophage Inflammatory protein-1; HIV for human immunodeficiency virus; P-388 for cancer cell line; c-MET for Mesenchymal–Epithelial Transition factor.

Organism	Group	Compound/Toxin	Activities	References
*Anthoptilum grandiflorum* (Antarctic Sea Pen)	Pennatulacea (sea pens)	Briarane diterpenes (bathyptilone A)	Cytotoxicity against the pluripotent embryonal carcinoma cell line, NTera-2 (NT2), isolated from lung metastasis of testicular cancer.	[105]
*Acabaria undulata*	Gorgonians (sea fans)	Steroids (7*α*,8*α*-epoxy-3*β*,5*α*,6*α*-trihydroxycholestane and 24-methyl-7*α*,8*α*-epoxy-3*β*,5*α*,6*α*-trihydroxycholest-22-ene)	Inhibitory effects towards phospholipases A2 (PLA2)	[106]
*Actinia equina*	Sea anemones	Na + channel neurotoxin (AE1)	Cytotoxic activity against mammalian red blood cells	[107]
*Actinia equina*	Sea anemones	Equinatoxin 11 (EqT II)	Hemolytic activity in rats	[108]
*Actinia equina*	Sea anemones	Equinatoxin III	Cardiovascular activity	[109]
*Actinia equina*	Sea anemones	Polypeptide toxin (Ae I)	Lethality against crabs	[96]
*Actinia equina*	Sea anemones	AEPI-I, II, III, and IV	Inhibition of serine proteases, trypsin, and α-chymotrypsin	[110]
*Actinia equina*	Sea anemones	Delta-actitoxin-Aeq2a/Ae 1 (Neurotoxin)	Type-1 sodium channel inhibitory activity	[95]
*Actinostola faeculenta*	Sea anemones	Extract	Cytotoxic effects against murine splenocytes and Ehrlich carcinoma cells	[100]
*Anemonia sulcata*	Sea anemones	Toxin I	Cardiotoxic activity	[111]
*Anemonia sulcata*	Sea anemones	Kalicludines and Kaliseptine	Blockers of voltage-sensitive K+ channels	[112]
*Anemonia viridis*	Sea anemones	Peptide toxin Av3	Lethality in Crustaceans	[113]
*Antheopsis maculata*	Sea anemones	Am I, II, III	Lethal activity against freshwater crabs (*Potamon dehaani*)	[114]
*Anthopleura aff. xanthogrammica*	Sea anemones	Kunitz-Type Protease inhibitors (AXPI-I and -II)	Inhibition of Trypsin and inhibition of other serine proteases (α-chymotrypsin and elastase (in case of AXPI-I only))	[115]
*Anthopleura asiatica*	Sea anemones	Bandaporin	Hemolytic activity in sheep red blood cells, lethal toxicity to crayfish	[116]
*Anthopleura elegantissima*	Sea anemones	Isotoxin APE 2-1	Cariotoxic effects in isolated right atria (guinea pig)	[115]
*Anthopleura elegantissima* and *Anthopleura nigrescens*	Sea anemones	Crude venom/extracts	Antimicrobial activities against human pathogens	[117,118]
*Anthopleura xanthogrammica*	Sea anemones	Sodium channel toxins	Enhancing sodium uptake in RT4-B and N1E-115 cells	[119]
*Anthopleura. xanthogrammica*	Sea anemones	Anthopleurin-A (AP-A) and Anthopleurin-B (AP-B)	Cardiotonic activity	[120]
*Antipathes dichotoma*	Black corals	Sphingolipids (ceramides)	Cytotoxicity against HepG2 (Hepatocellular carcinoma), WI 38 (Normal human embryonic lung fibroblasts), VERO (Normal kidney epithelial cells), and MCF-7 (Human breast adenocarcinoma).	[121]
*Antipathes dichotoma*	Black corals	Steryl Esters/steryl hexadecanoates (3β-hexadecanoylcholest-5-en-7-one) and Thymidine	Anticancer activity against VERO and MCF-7	[122]
*Asterospicularia laurae*	Soft corals	Asterolaurins A−F	Inducing moderate cytotoxicity against HepG2 cells and inhibition of elastase release and superoxide anion generation	[123]
*Astrogorgia* sp.	Gorgonians (sea fans)	Calicoferol A and E, 24-exomethylenecalicoferol E, 9β-hydroxy-9,10-secosteroid astrogorgol F, and 9α-hydroxy-9,10-secosteroid astrogorgi-adiol	Suppression of tumor-associated kinases	[124]
*Bartholomea annulata*	Sea anemones	Polypeptide	Neurotoxic activity in sea crabs (Ocypode quadrata)	[125]
*Briareum excavatum*	Soft corals	Briaexcavatins	Mild cytotoxicity toward MDA-MB-231 human breast tumor cells and inhibiting neutrophil elastase release in humans	[126]
*Briareum excavatum*.	Soft corals	Briaexcavatolides	Cytotoxicity towards cancer cell lines	[127]
*Briareum polyanthes*	Soft corals	Briarellins and polyanthellin A	Antimalarial activity against *Plasmodium falciparum.*	[128]
*Bunodosoma caissarum*	Sea anemones	Caissarolysin I (Bcs I)	Hemolytic activity to human erythrocytes	[94]
*Bunodosoma caissarum*	Sea anemones	BcsTx3 toxin	Potassium channel blocker	[129]
*Bunodosoma caissarum*	Sea anemones	BcI, II, and II	Neurotoxicity and hemolytic activity	[130]
*Bunodosoma caissarum*	Sea anemones	PLA2 proteins (BcPLA21, BcPLA22, and BcPLA23	Inducing insulin secretion in the presence of high glucose, increasing perfusion pressure, renal vascular resistance, urinary flow, glomerular filtration rate, and sodium, potassium, and chloride levels of excretion in isolated kidney	[131]
*Bunodosoma cangicum.*	Sea anemones	Cangitoxin (CGTX-II and CGTX-III)	Sodium (Nav1.1) channels toxin	[132]
*Bunodosoma granulifera*	Sea anemones	Granulitoxin (GRX)	Severe neurologic effects such as aggressive behavior, dyspnea, circular movements, etc.	[133]
*Bunodosorna granulifera*	Sea anemones	Peptide toxin	Facilitating acetylcholine release at avian neuromuscular junctions, competing with dendrotoxin I for attachment to synaptosomal membranes of rat brain, and suppressing potassium currents in rat dorsal root ganglion neurons in culture.	[134]
*Calliactis parasitica*	Sea anemones	Calitoxin (CLX)	Inducing a strong release of neurotransmitters, which causes high muscle contraction in crustaceans	[135]
*Capnella imbricata* (Formosan Soft Coral)	Soft corals	Capnellenes (sesquiterpenes)	Anti-inflammatory activity	[136]
*Cespitularia hypotentaculata*	Soft corals	Cespitularins	Cytotoxicity against cancer cell lines	[137]
*Clavularia koellikeri*	Soft corals	Marine diterpenoid	Cytotoxic activity against adenocarcinoma cells (DLD-1) and potent growth inhibiting activity against human T lymphocytic leukemia cells (MOLT-4)	[138]
*Clavularia* sp.,	Soft corals	Stolonidiol (Diterpenoid)	Producing a neurotrophic factor-like agent on the cholinergic nervous system	[139]
*Clavularia viridis*	Soft corals	Marine prostanoids (claviridic acids A–E)	Inhibitory effect on PHA-induced proliferation of peripheral blood mononuclear cells (PBMC) and cytotoxicity against human gastric cancer cells (AGS)	[140]
*Clavularia viridis*	Soft corals	Bromovulone III and chlorovulone II	cytotoxicity against human prostate (PC-3) and colon (HT29) cancer cells	[141]
*Clavularia viridis*	Soft corals	Marine prostanoid, bromovulone III	Antitumor activity in human hepatocellular carcinoma	[141]
*Clavularia viridis*	Soft corals	Chlorinated marine steroids (Yonarasterols)	Antitumor activity	[138]
*Condylactis gigantea*	Sea anemones	Peptide toxins (type I sea anemone sodium channel toxins)	Paralytic activity on crab	[142]
*Condylactis gigantea*	Sea anemones	Phospholipase A2(CgPLA2)	Hemolytic activity, enzymatic activity	[143]
*Condylactis gigantea*	Sea anemones	Peptide toxin, CgNa	Type I sodium channel toxin, prolonging the duration of cardiac activity, and enhancing contractile force in rats	[142]
*Condylactis gigantea*	Sea anemones	Phospholipase A2 (CgPLA2)	High catalytic activity upon fluorescent phospholipids	[143]
*Condylactis passiflora*	Sea anemones	Polypeptide toxins (Cp I, II, and III)	Lethal activity against crabs	[144]
*Corallimorphus* cf. *pilatus*	Corallimorphs	Extract	Antibacterial and antifungal activities	[100]
*Cribrinopsis similis*	Sea anemones	Bioactive Polypeptides	Hemolytic activity, Laminarinase activity	[145]
*Dendronephthya rubeola*	Soft corals	Capnellenes (dihydroxycapnellene (capnell-9(12)-ene-8β,10α-diol))	Antiproliferative (against murine fibroblast (L-929) cell line) and Cytotoxic (against Human cervix carcinoma (HeLa) cell line) activity, Inhibition of the Tax/CREB, Myc/Max, and Myc/Mic-1 interaction	[146]
*Dendronephthya* sp.,	Soft corals	Sogosterones A−D	Antifouling activities	[147]
*Elesto riisei*	Gorgonians (sea fans)	Punaglandins	Inhibiting Ubiquitin isopeptidase activity and exhibiting antiproliferative effects	[148]
*Eunicea fusca*	Gorgonians (sea fans)	Fuscosides	Anti-inflammatory activities	[149]
*Eunicea knight*	Gorgonians (sea fans)	Cembranes asperdiol	Inhibitory activity against PTP1B	[150]
*Eunicea* sp.,	Gorgonians (sea fans)	Sesquiterpenes (elemane, eudesmane, and germacrane types)	Inhibitory effect on the growth of the malarial parasite *Plasmodium falciparum*	[151]
*Euplexaura anastomosans*	Gorgonians (sea fans)	Farnesylhydroquinone glycosides (Euplexides), and Euplexide A, B, and G	Inhibition of Phospholipases A2 (PLA2)	[152]
*Euplexaura robusta*	Gorgonians (sea fans)	Tetra-prenylated alkaloid, Malonganenone D	Inhibitory activity against MET Receptor Tyrosine Kinase, or c-Met	[153]
Fungus *Scopulariopsis* sp., isolated from the inner tissue of the coral *Stylophora*	Stony corals (Host)	Antibiotic AGI-B4, violaceol I, violaceol II, scopularide A	Cytotoxicity against mouse lymphoma cell line (L5178Y)	[81]
Fungus *Scopulariopsis* sp., isolated from the inner tissue of the coral *Stylophora*	Stony corals (Host)	3β,7β,15α,24-tetrahydroxyolean-12-ene-11,22-dione and 15α,22β,24-trihydroxyolean-11,13-diene-3-one	Cytotoxicity against mouse lymphoma cell line (L5178Y)	[84]
*Gorgonian Pseudopterogorgia* sp.	Gorgonians (sea fans)	9,11-secosterol	Inhibitory effects on Protein kinase C (PKC)/Antiproliferative potential	[154]
*Halcurias* sp.	Sea anemones	Halcurin	Lethality against crabs	[155]
*Heteractis crispa*	Sea anemones	Kunitz/BPTI-type peptides	Neuroprotective activity against 6-hydroxydopamine-induced neurotoxicity.	[156]
*Heteractis crispa*	Sea anemones	Polypeptide toxin (π-AnmTX Hcr 1b-1)	Inhibiting the ASIC3 acid-sensitive channel	[157]
*Heteractis crispa*	Sea anemones	ASIC1a inhibitor (Hcr 1b-2)	Antihyperalgesic effects in the acid-induced pain model	[158]
*Heteractis crispa*	Sea anemones	Kunitz-type inhibitors/polypeptides (HCRG1 and HCRG2)	Anti-inflammatory	[159]
*Heteractis crispa*	Sea anemones	HCRG21	Inhibiting the capsaicin-induced current through the transient receptor potential family member vanilloid 1 (TRPV1)	[160]
*Heteractis crispa*	Sea anemones	Polypeptides (APHC2 and APHC3)	Analgesic effect on mammals	[161]
*Heteractis crispa*	Sea anemones	Recombinant polypeptide (HCGS 1.20)	Anti-inflammatory activity	[162,163]
*Heteractis magnifica*	Sea anemones	Cytolysin, HMgIII	Hemolytic activity	[164]
*Heteractis magnifica*	Sea anemones	HMIQ3c1 recombinant peptide	Neuroprotective activity in a model of Alzheimer’s disease.	[165]
*Heteractis magnifica*	Sea anemones	Magnificamide, a β-Defensin-Like Peptide	Inhibition of α-amylases (treatment of type 2 diabetes mellitus)	[166]
*Heteractis magnifica (formerly Radianthus ritteri)*	Sea anemones	Magnificalysins I and II	Hemolytic activities and lethal effects	[167]
*Heteractis magnifica.*	Sea anemones	Potassium channel toxin, HmK	Inhibiting the binding ofα-dendrotoxin (a ligand for voltage-gated K channels) to rat brain synaptosomal membranes, blocking K+ currents through Kv 1.2 channels expressed in a mammalian cell line, and facilitating acetylcholine release at the avian neuromuscular junction	[168]
*Isis hippuris*	Gorgonians (sea fans)	Suberosane sesquiterpenes (suberosenol B, suberosanone, suberosenol B acetate)	Exhibiting potent cytotoxicity toward P-388, A549, and HT-29 cancer cell lines	[169]
*Isis hippuris*	Gorgonians (sea fans)	Isishippuric acid B	Producing potent cytotoxicity toward a limited panel of cancer cells	[170]
*Isis hippuris*	Gorgonians (sea fans)	Hippuristanols	Cytotoxicity against several cancer cell lines.	[171]
*Isis minorbrachyblasta*	Gorgonians (sea fans)	22-epihippuristanol and hippuristanol	Cytotoxicity against cancer cell lines (A549, HONE1, HeLa)	[172]
*Junceella fragilis*	Gorgonians (sea fans)	Juncin Z	Anti-inflammatory activity	[173]
*Junceella fragilis*	Gorgonians (sea fans)	Briarane-type diterpenoids (Frajunolides)	Antioxidant activities	[174]
*Junceella juncea*	Gorgonians (sea fans)	Junceoal A	Inhibition of superoxide anions by human neutrophils	[175]
*Junceella juncea*	Gorgonians (sea fans)	Juncin ZII	Antifouling activity against the larval settlement of barnacle *Balanus Amphitrite* and antifeedant activity against second-instar larvae of *Spodoptera litura*	[176]
*Klyxum simplex*	Soft corals	Klysimplexins B and H	Cytoxic activity in human cancer cells (liver carcinoma, gingival carcinoma, breast cancer)	[177]
*Klyxum simplex*	Soft corals	Simplexin E	Reduction of iNOS (Inducible nitric oxide synthase) and COX-2 (cyclooxygenase-2) proteins in lipo-polysaccharide (LPS)-stimulated macrophage cells	[178]
*Liponema brevicorne, Actinostola callosa*	Sea anemones	Extracts	Hemolytic activity	[100]
*Lobophytum crassum*	Soft corals	Crassumolides	Anti-inflammatory (inhibits the accumulation of the pro-inflammatory proteins iNOS and COX-2) and cytotoxic activities	[179]
*Lobophytum cristagalli*	Soft corals	Cembranolide diterpene	Inhibition of farnesyl protein transferase (FPT)	[180]
*Lobophytum cristagalli*	Soft corals	Cembranolide (diterpene)	Inhibition of farnesyl protein transferase (FPT)	[180]
*Lobophytum durum*	Soft corals	Durumolides	Anti-inflammatory effects and antibacterial activities	[181]
*Lobophytum durum*	Soft corals	Durumhemiketalolides	Anti-inflammatory activities	[182]
*Lobophytum* Species	Soft corals	Cembranoid diterpenes (Lobohedleolide, (7*Z)* -lobohedleolid, 17-Dimethylaminolobohedleolide)	HIV inhibitory activity	[183]
*Lobophytum* Species	Soft corals	Lobophytene	Cytotoxic activities against lung (A549) and colon (HT-29) cell lines	[184]
Marine actinomycetes associated with stony corals	Stony corals (Host)	Bioactive metabolites	Antimalarial, antibacterial, antifungal, antimicrobial, anti-inflammatory, cytotoxic, and antitumor activity	[185]
Marine bacterium *Erythrobacter flavus* strain KJ5 from hard coral *Acropora nasuta*	Stony corals	Sulfotransferases	Antithrombotic, antifouling, antiviral, and anti-inflammatory activities	[87,88]
Marine-derived fungus *Gliomastix* sp., from *Stylophora* sp.	Stony corals (Host)	Gliomastins A–D, 9-O-methylgliomastin C, acremonin A 1-O-β-D-glucopyranoside, gliomastin E 1-O-β-D-glucopyranoside, and 6′-O-acetyl-isohomoarbutin.	Cytotoxicity against mouse lymphoma cell line (L5178Y)	[85]
*Metridium senile*	Sea anemones	Peptide, τ-AnmTX Ms 9a-1 (short name: Ms 9a-1)	Anti-inflammatory and analgesic effects in mice	[186]
*Nephthea chabroli*	Soft corals	Chabranol (terpenoid)	Cytotoxicity against mouse lymphocytic leukemia (P-388) cell line	[187]
*Nephthea erecta*	Soft corals	Oxygenated ergostanoids	Anti-inflammatory effects	[188]
*Oulactis orientalis*	Sea anemones	Cytolysins Or-A and Or-G	Hemolytic activity	[189]
*Palythoa caribaeorum*	Zoanthids	Nematocyst Venom	Antitumor activity (human glioblastoma (U251) and (human lung adenocarcinoma (SKLU-1) cell lines), Antigardial activity against the parasite *Giardia intestinalis*	[190]
*Palythoa caribaeorum*	Zoanthids	Phospholipase (A2-PLTX-Pcb1a)	Neurotoxic activity in rats’ tissues.	[191]
*Palythoa. caribaeorum*	Zoanthids	PcKuz3 isotoxin	Neuroprotective activity	[65]
*Paramuricea* sp., (Deep-sea gorgonian)	Gorgonians (sea fans)	Linderazulene	Moderate cytotoxicity against P388 murine leukemia cell line	[192]
*Parazoanthus axinellae*	Zoanthids	cnidocyst extract	Antimicrobial activities (human and aquaculture)	[66]
*Phyllodiscus semoni*	Sea anemones	PsTX-60A and PsTX-60B	Lethal activity to shrimp *Palaemon paucidence*, hemolytic activity on sheep red blood cells	[193]
*Phyllodiscus semoni*	Sea anemones	Hemolytic toxins—Pstx20	Hemolytic activity	[194]
*Phyllodiscus semoni*	Sea anemones	PsTX-T	Nephrotoxic activity (glomerular endothelial damage)	[195]
*Phyllodiscus semoni*	Sea anemones	PsTX-60A and PsTX-60B	Lethal toxicity to shrimp *Palaemon paucidence*, hemolytic activity towards sheep erythrocytes	[193]
*Phymanthus crucifer*	Sea anemones	Toxin, PhcrTx1	Inhibiting acid-sensing ion channel (ASIC)	[196]
*Pinnigorgia* sp.,	Gorgonians (sea fans)	Pinnigorgiols A-E	Anti-inflammatory activity	[197]
*Pinnigorgia* sp.,	Gorgonians (sea fans)	Apo-9′-fucoxanthinone	Inhibition of elastase release by human neutrophils	[198,199]
*Pocillopora damicornis* associated fungus, *Acremonium sclerotigenum*	Stony corals (host)	4-hydroxy-2-pyridone alkaloid and phenazine alkaloid	Cytotoxicity against two prostate cancer cell lines, anti-Vibrio activity	[200]
*Porites astreoides*	Stony corals	Aqueous extract	Hemolytic activity in humans and rats	[77]
*Protopalythoa variabilis*	Zoanthids	Lipidic α-amino acids/LAAs	Cytotoxic activity against human tumor cell lines: HCT-8 (human colon carcinoma), MDA-MB-435 (melanoma), SF-295 (CNS glioblastoma), and HL-60 (leukemia)	[201]
*Pseudodiploria strigosa*	Stony corals	Aqueous extract	Hemolytic activity in humans and rats	[77]
*Pseudoplexaura porosa*	Gorgonians (sea fans)	14-acetoxycrassine	Inhibitory activity against PTP1B	[150]
*Pseudopterogogia* *elisabethae*	Gorgonians (sea fans)	Elisapterosin B	In vitro antituberculosis activity	[202]
*Pseudopterogogia* *elisabethae*	Gorgonians (sea fans)	Aberrarone	In vitro antimalarial activity against a chloroquine-resistant strain of the protozoan parasite *Plasmodium falciparum*	[203]
*Pseudopterogogia elisabethae*	Gorgonians (sea fans)	Methanol extract	Antibacterial activity selectively against the *Gram*-positive bacteria *Streptococcus pyogenes, Staphylococcus aureus*, and *Enterococcus faecalis*	[204]
*Pseudopterogogia elisabethae*	Gorgonians (sea fans)	Homopseudopteroxazole	Strong growth inhibitor of *Mycobacterium tuberculosis*	[205]
*Pseudopterogorgia acerosa*	Gorgonians (sea fans)	lipidyl pseudopteranes A and D	Inhibitory activity against PTP1B (Protein tyrosine phosphatase 1B)	[206]
*Pseudopterogorgia acerosa*	Gorgonians (sea fans)	Bis(pseudopterane) amine	Growth inhibition activity against cancer cell lines (HCT116 and HeLa)	[207]
*Pseudopterogorgia bipinnata*	Gorgonians (sea fans)	Bipinnapterolide B	Antitubercular activity	[103,104]
*Pseudopterogorgia bipinnata*	Gorgonians (sea fans)	Caucanolides A−F	In vitro antiplasmodial activity against the malaria parasite, *Plasmodium falciparum*	[208]
*Pseudopterogorgia elisabethae*	Gorgonians (sea fans)	Carienol A and B, Elisapterosin B	Antitubercular activity	[103,104]
*Pseudopterogorgia elisabethae*	Gorgonians (sea fans)	Ileabethoxazole	Inhibition of *Mycobacterium tuberculosis*	[125]
*Pseudopterogorgia kallos*	Gorgonians (sea fans)	Bielschowskysin	Antimalarial activity against *Plasmodium falciparum*, as well as strong anticancer activity against human cancer cell lines	[209]
*Pseudopterogorgia rigida*	Gorgonians (sea fans)	Curcuphenol	Antibacterial activity	[210]
*Pseudopterogorgia. elisabethae* and its dinoflagellate (*Symbiodinium* sp.) symbiont	Pennatulacea (Sea fans (Host)	Pseudopterosin X and Y (diterpenes)	Re-epithelialization and enhanced wound healing	[103,204]
*Pseudoterogorgia rigida*	Gorgonians (sea fans)	Perezone	Cytotoxicity against human cancer cell lines	[211]
*Radianthus crispus*	Sea anemones	Polypeptide toxin (Re I)	Lethality against crabs	[212]
*Radianthus macrodactylus*	Sea anemones	Actinoporin RTX-S II	Hemolytic activity and lethal effects	[213]
*Sarcophyton crassocaule*	Soft corals	Polyoxygenated cembranoids ((crassocolides G–M,) Crassocolides N-P) Crassocolide N	Cytotoxicity against human medulloblastoma (Daoy cells), human oral epidermoid carcinoma, and human cervical epithelioid carcinoma cell lines	[214,215]
*Siderastrea siderea*	Stony corals	Aqueous extract	Hemolytic activity in humans and rats	[77]
*Sinularia flexibilis*	Soft corals	Flexilarin D	Exhibiting cytotoxicity against Hep2 tumor cells	[216]
*Sinularia flexibilis*	Soft corals	11-episinulariolide	Exhibiting strong algacidal properties	[217]
*Sinularia gibberosa*	Soft corals	Gibberoketosterol	Anti-inflammatory effects and cytotoxicity	[218]
*Sinularia gibberosa* and *Sarcophyton trocheliophorum*	Soft corals	Cembranoids Diterpenes	Cytotoxic effects	[219]
*Sinularia polydactyla*	Soft corals	Methanol and hexane extracts	Inhibition of biofilm-forming bacteria	[220]
*Sinularia querciformis*	Soft corals	Querciformolide C	Anti-inflammatory effects	[221]
*Stichodactyla gigantea*	Sea anemones	Gigantoxins I	Exhibiting human epidermal growth factor (EGF) like activity	[222]
*Stichodactyla haddoni*-associated bacteria	Sea anemones (Host)	Culture extracts	Antimicrobial activity, human bacterial and fungal pathogens	[223]
*Stichodactyla helianthus*	Sea anemones	ShK	Inhibiting voltage-dependent potassium channels, competing with dendrotoxin I and α-dendrotoxin for attachment to synaptosomal membranes of rat brain, and suppressing potassium currents in rat dorsal root ganglion neurons in culture.	[224]
*Stichodactyla helianthus*	Sea anemones	Helianthamide (β-Defensin-like Protein)	Inhibiting glycosidase (controlling blood sugar levels in the management of diabetes)	[225]
*Stichodactyla helianthus*	Sea anemones	Sticholysin I (St-I) and sticholysin II (St-II)	Hemolytic activity	[226]
*Streptomyces* sp. SCSIO 41399, from coral *Porites* sp.	Stony corals (host)	Isotirandamycin B, tirandamycin A, and tirandamycin B	Bacteriostatic activity against *Streptococcus agalactiae*	[89]
*Stylophora* sp.	Stony corals	Δ-Pocilopotoxin-Spi1 (Δ-PCTX-Spi1	Hemolytic activity	[227]
*Subergorgia* sp.,	Gorgonians (sea fans)	Astaxanthin	Inhibitory activity towards the IKKbeta kinase	[228]
*Tubastraea coccinea* and *Tubastraea tagusensis* (Sun corals)	Stony corals	Extracts	Anti-inflammatory activity, Cytotoxicity	[229]
*Urticina aff. coriacea*		Biologically active peptides	Inhibiting currents of mammalian ASIC1a channels	[230]
*Urticina crassicornis*	Sea anemones	UcI (Cytolysin)	Cytolytic activity	[231]
*Urticina eques*	Sea anemones	Peptide-τ-AnmTx Ueq 12-1	Antibacterial against Gram-positive bacteria and a potentiating activity on the transient receptor potential ankyrin 1 (TRPA1)	[186]
*Urticina grebelnyi*	Sea anemones	π-AnmTX Ugr 9a-1 (Ugr 9-1)	Anti-inflammatory activity and reversing acid-induced pain using in vivo mice	[232]
*Urticina piscivora*	Sea anemones	UpI protein	Hemolytic activity on erythrocytes of rat, guinea pig, dog, pig, and human	[233]
*Veretillum malayense*	Pennatulacea (sea pens)	Briarane diterpenes (Malayenolides A−D), Malayenolide A	Toxic to brine shrimp	[234]
*Virgularia gustaviana*	Pennatulacea (sea pens)	Cholest,5en,3ol (cholesterol), Hexadecanoic acid, 2-Hexadecanol	Reduced viability of breast cancer cell line MDA-MB-231 and human cervical cancer cell line HeLa, induction of apoptosis.	[235]
*Virgularia juncea*	Pennatulacea (Sea pens)	Sesquiterpenoid (Junceol A) and diterpenoids (Sclerophytin A and Cladiellisin.	Cytotoxicity towards P-388 cancer cells	[236]
*Xenia blumi*	Soft corals	Blumiolide C (diterpenoid)	Cytotoxicity against mouse lymphocytic leukemia cells (P-388)	[237]
*Xenia novaebrittanniae*	Soft corals	Xeniolides I	Antibacterial activity	[238]
*Xenia novaebrittanniae*	Soft corals	Novaxenicins (diterpenoids)	Pro-apoptotic activity in transformed mammalian cells	[238]
*Zoanthus cf.pulchellus*	Zoanthids	Zoanthamine	ROS and NO modulators in Neuroinflammation in microglia BV-2 cells	[239]
*Zoanthus sociatus*	Zoanthids	Y-like polypeptide, ZoaNPY	Proangiogenic activity (In vitro)	[63]
*Zoanthus. natalensis*	Zoanthids	ZoaKuz1	Neuroprotective activity	[64]

**Table 2:** This table reports the genomes available on the public GenBank database (updated on 7 April 2025) annotated by NCBI RefSeq or by GenBank submitter.

Assembly Accession	Organism Name	Assembly Stats: Total Sequence Length	Assembly Release Date	Assembly Sequencing Tech	Annotation Count Gene Total	Annotation Count Gene Protein-coding
GCA_932526225.1	*Nematostella vectensis*	269418438	23 March 2022	PacBio, (Pacific Biosciences, Menlo Park, CA, USA) and Arima2 (Arima Genomics, Inc., San Diego, CA, USA)	38762	19231
GCA_013753865.1	*Acropora millepora*	475381253	29 July 2020	PacBio (Pacific Biosciences, Menlo Park, CA, USA)	42775	30136
GCA_036669905.1	*Acropora muricata*	487333246	22 February 2024	PacBio Sequel II (Pacific Biosciences, Menlo Park, CA, USA)	44841	26093
GCA_036669915.2	*Pocillopora verrucosa*	353416094	22 February 2024	PacBio Sequel II (Pacific Biosciences, Menlo Park, CA, USA)	32915	25867
GCA_036669935.2	*Montipora foliosa*	789253072	23 August 2024	PacBio Sequel II (Pacific Biosciences, Menlo Park, CA, USA)	46203	31642
GCA_036669925.2	*Montipora capricornis*	809264945	23 August 2024	PacBio Sequel II (Pacific Biosciences, Menlo Park, CA, USA)	47382	32425
GCA_001417965.1	*Exaiptasia diaphana*	256132296	28 October 2015	Illumina HiSeq; Illumina MiSeq (Illumina, Inc., San Diego CA, USA)	26789	26042
GCA_001417965.1	*Exaiptasia diaphana*	256132296	28 October 2015	Illumina HiSeq; Illumina MiSeq (Illumina, Inc., San Diego, CA, USA)	24862	22509
GCA_000222465.2	*Acropora digitifera*	447478678	15 January 2016	454 GS FLX (Roche (via Roche Applied Science), Basel, Switzerland); Illumina Genome Analyzer (Illumina, Inc., San Diego, CA, USA)	32106	26073
GCA_002571385.2	*Stylophora pistillata*	397587675	17 October 2017	Illumina HiSeq (Illumina, Inc., San Diego, CA, USA)	25506	23941
GCA_002571385.2	*Stylophora pistillata*	397587675	17 October 2017	Illumina HiSeq (Illumina, Inc., San Diego, CA, USA)	28498	24473
GCA_002042975.1	*Orbicella faveolata*	485532801	20 March 2017	Illumina HiSeq; Illumina MiSeq (Illumina, Inc., San Diego, CA, USA)	30178	25929
GCA_003704095.1	*Pocillopora damicornis*	234333463	31 October 2018	Illumina (Illumina, Inc., San Diego, CA, USA)	26075	25422
GCA_003704095.1	*Pocillopora damicornis*	234333463	31 October 2018	Illumina (Illumina, Inc., San Diego, CA, USA)	23077	19935
GCA_029204205.1	*Desmophyllum pertusum*	556858542	8 March 2023	PacBio Sequel (Pacific Biosciences, Menlo Park, CA, USA); Illumina HiSeq (Illumina, Inc., San Diego, CA, USA)	40679	37484
GCA_009602425.1	*Actinia tenebrosa*	238179736	6 November 2019	Illumina HiSeq (Illumina, Inc., San Diego, CA, USA)	22927	19980
GCA_942486035.1	*Porites lobata*	646152978	15 May 2023	ONT (Oxford Nanopore Technologies Ltd., Oxford, UK;), Illumina (Illumina, Inc., San Diego, CA, USA)	42872	42872
GCA_004324835.1	*Dendronephthya gigantea*	286131786	4 March 2019	PacBio (Pacific Biosciences, Menlo Park, CA, USA)	29721	22045
GCA_902702795.2	*Paramuricea clavata*	606969498	8 November 2022	Illumina (Illumina, Inc., San Diego, CA, USA); ONT (Oxford Nanopore Technologies Ltd., Oxford, UK).	1600035	62650
GCA_942486045.1	*Pocillopora meandrina*	347233126	15 May 2023	ONT (Oxford Nanopore Technologies Ltd., Oxford, UK); Illumina (Illumina, Inc., San Diego, CA, USA).	32095	32095
GCA_942486025.1	*Porites evermanni*	603805388	15 May 2023	Illumina (Illumina, Inc., San Diego, CA, USA)	40381	40380
GCA_032359415.1	*Acropora cervicornis*	307445771	4 October 2023	Oxford Nanopore MinION (Oxford Nanopore Technologies Ltd., Oxford, UK); Illumina HiSeq (Illumina, Inc., San Diego, CA, USA)	33794	28059
GCA_021976095.1	*Xenia* sp. *Carnegie-2017*	222699550	4 February 2022	Illumina (Illumina, Inc., San Diego, CA, USA); Nanopore (Oxford Nanopore Technologies Ltd., Oxford, UK); Hi-C (Arima Genomics, Inc., San Diego, CA, USA)	26389	18425
GCA_033675265.1	*Actinostola* sp. *cb2023*	424251646	15 November 2023	PacBio Sequel (Pacific Biosciences, Menlo Park, CA, USA)	20812	20812
GCA_000209225.1	*Nematostella vectensis*	356613585	22 August 2007	-	27173	24773
GCA_000209225.1	*Nematostella vectensis*	356613585	22 August 2007	-	37751	23845
GCA_004143615.1	*Acropora millepora*	386599652	6 February 2019	Illumina HiSeq (Illumina, Inc., San Diego, CA, USA).	31132	23710
GCA_030620025.1	*Pocillopora verrucosa*	356884395	3 August 2023	Oxford Nanopore PromethION (Oxford Nanopore Technologies Ltd., Oxford, UK); Illumina NovaSeq (Illumina, Inc., San Diego, CA, USA)	32047	26915

## Data Availability

Not applicable.
